# Cathepsin S inhibition combines control of systemic and peripheral pathomechanisms of autoimmune tissue injury

**DOI:** 10.1038/s41598-017-01894-y

**Published:** 2017-06-05

**Authors:** Maia Tato, Santhosh V. Kumar, Yajuan Liu, Shrikant R. Mulay, Solange Moll, Bastian Popper, Jonathan N. Eberhard, Dana Thomasova, Arne Christian Rufer, Sabine Gruner, Wolfgang Haap, Guido Hartmann, Hans-Joachim Anders

**Affiliations:** 1 Medizinische Klinik and Poliklinik IV, Renal Division, Klinikum der Universität München, Campus Innenstadt, München, Germany; 2 Dalian Central Hospital, Southern Medical University, Dalian, China; 3 Division of Clinical Pathology, Department of Pathology and Immunology, University Hospital Geneva, Geneva, Switzerland; 4 Department of Anatomy and Cell Biology, Biomedical Center, Ludwig-Maximilians Universität, Planegg-Martinsried, Germany; 5 Roche Innovation Centre Basel, Pharma Research and Early Development, Hoffmann La Roche, Basel, Switzerland

## Abstract

Cathepsin(Cat)-S processing of the invariant chain-MHC-II complex inside antigen presenting cells is a central pathomechanism of autoimmune-diseases. Additionally, Cat-S is released by activated-myeloid cells and was recently described to activate protease-activated-receptor-(PAR)-2 in extracellular compartments. We hypothesized that Cat-S blockade targets both mechanisms and elicits synergistic therapeutic effects on autoimmune tissue injury. MRL-(Fas)lpr mice with spontaneous autoimmune tissue injury were treated with different doses of Cat-S inhibitor RO5459072, mycophenolate mofetil or vehicle. Further, female MRL-(Fas)lpr mice were injected with recombinant Cat-S with/without concomitant Cat-S or PAR-2 blockade. Cat-S blockade dose-dependently reversed aberrant systemic autoimmunity, e.g. plasma cytokines, activation of myeloid cells and hypergammaglobulinemia. Especially IgG autoantibody production was suppressed. Of note (MHC-II-independent) IgM were unaffected by Cat-S blockade while they were suppressed by MMF. Cat-S blockade dose-dependently suppressed immune-complex glomerulonephritis together with a profound and early effect on proteinuria, which was not shared by MMF. In fact, intravenous Cat-S injection induced severe glomerular endothelial injury and albuminuria, which was entirely prevented by Cat-S or PAR-2 blockade. *In-vitro* studies confirm that Cat-S induces endothelial activation and injury via PAR-2. Therapeutic Cat-S blockade suppresses systemic and peripheral pathomechanisms of autoimmune tissue injury, hence, Cat-S is a promising therapeutic target in lupus nephritis.

## Introduction

Autoimmune diseases present in many different facets depending on the tissue distribution of the respective autoantigens. However, behind the variety of clinical presentations autoimmune diseases are driven by a releatively homogenous activation of the innate and adaptive immune system^1^. Before having fully understood the molecular and cellular complexity of such immune responses non-specific immunosuppressants such as steroids, cyclophosphamide, and mycophenolate mofetil (MMF) were found to be effective in suppressing systemic symptoms and tissue manifestations of autoimmune disease. The non-selective nature of such drugs explains their broad toxicity profiles^2^. Thus, it remains necessary to develop more specific drugs to control autoimmune disease. Specific targeting of immune processes has become possible with biological drugs, some of which have proven to be extremely efficent in controling autoimmune diseases such as anti-TNF-α in rheumatoid arthritis and Crohn’s disease or anti-CD20 in rheumatoid arthritis and ANCA vasculitis. However, these drugs are less effective in other forms of systemic autoimmunity^3, 4^, probably because they may not target universal pathomechanisms of autoimmunity.

A central and non-redundant element for the activation of autoantigen-specific immunity is major histocompatibility complex (MHC) class II-mediated autoantigen presentation^5^. Cathepsin-S (Cat-S) is a cysteine protease of the papain family inside lysosomal/endosomal compartments of antigen-presenting cells, such as B cells, macrophages and dendritic cells^6^. Inside the B cells and dendritic cells, Cat S is the single enzyme that cleaves the lip10^5^p10^5^; a 10-kDa fragment of the MHC-II bound invariant chain that forms the 24 amino-acid CLIP fragments during the assembly of the MHC class II-α and -β chains with the antigenic peptide in the lysosomal/endosomal compartments^7–13^. In addition, Cat-S limits auto-reactive CD4 T cell escape from thymic selection by degrading auto-antigenic peptides^14^, hence, there is a robust rationale for Cat-S being a mediator of autoimmunity. Lack of Cat-S or Cat-S inhibition was shown to suppress autoimmunity in a number of animal models such as autoimmune encephalitis^15^, collagen-related autoimmunity^15^, Sjögren’s syndrome^16^ or SLE^17^.

We and others recently discovered that Cat-S has an additional biological effect, i.e. the proteolytic cleavage of protease-activated receptor (PAR)-2 on the surface of vascular endothelial cells, a process that contributes to microvascular complications in diabetes mellitus^18–21^. Therefore, we hypothesized that Cat-S inhibition may have a dual therapeutic effect on vascular autoimmune tissue injury, i.e. suppression of MHC-II-dependent systemic autoimmunity as well as peripheral tissue protection from autoimmune vascular injury. To address this concept we developed a novel highly-specific Cat-S inhibitor and tested it *in vivo* in comparison to standard immunosuppression in a model of lupus-like immune complex-related vasculopathy of the kidney, i.e. lupus nephritis.

## Results

### Pharmacodynamics and pharmacokinetics of RO5459072 in mice

To test the functional contribution of Cat-S in systemic autoimmunity we used the inhibitor RO5459072. RO5459072 is a potent and highly selective compound that inhibits human Cat-S with an apparent IC50 of 0.1 nM and murine Cat-S of 0.3 nM (Supplementary Table 1). No sub-micro molar inhibition was detected on any other of the tested cathepsins (Cat L, B, K, and F) with the exception of Cat V with apparent IC50 of 700 nM. In addition, RO5459072 showed ≤30% inhibition on a diversity panel consisting of nearly 100 receptor binding and enzymatic assays at 10 µM concentration (not shown). The ability of RO5459072 to inhibit Cat-S in cells was tested with the lip10 accumulation assay in human purified B-cells from whole blood and a potent induction of lip10 was determined with EC50 of 15.8 nM (Supplementary Figure 1A). Due to the high affinity and formation of a covalent bond between the nitrile warhead of RO5459072 and the catalytic cystein residue of Cat-S, the binding of the inhibitor is irreversible on the time scale of *in vitro* kinetic experiments. Thus, depletion of the concentrations of free inhibitor and free enzyme upon formation of the covalent complex need to be accounted for during quantitative analysis of the data using the Morrison equation. A value of Ki = 66 ± 25 pM was calculated for the inhibitor RO5459072 (Supplementary Figure 1B–D). The acute pharmacodynamic effect of RO5459072 *in vivo* was tested after oral gavage of doses from 0.1–100 mg/kg. Splenic induction of p10 was used as a measure of enzyme inhibition. A strong p10 up-regulation was detected with maximal induction at low doses of 1 mg/kg corresponding to a plasma concentration of the inhibitor of 17 nM (Supplementary Figure 1E). Oral administration of RO5459072 by food admix (262 mg/kg of food) to female MRL-(Fas)lpr mice resulted in an exposure dose of 30 mg/kg and stable plasma levels of RO5459072 at 400–600 ng/ml over a period of 8 weeks. Mean plasma concentrations (SD) were 172 ± 58.4, 648 ± 142 and 2660 ± 575 ng/ml at 3, 10 and 30 mg/kg/day respectively. This was associated with a robust p10 fragment accumulation in the spleen (Supplementary Figure 1E). In summary, RO5459072 is a specific small molecule Cat-S inhibitor with favorable pharmacodynamic and pharmacokinetic profiles to efficiently block Cat-S over prolonged periods of time in MRL-(Fas)lpr mice.

### Cathepsin S inhibition attenuates systemic autoimmunity in MRL-(Fas)lpr mice

**Figure 1:**
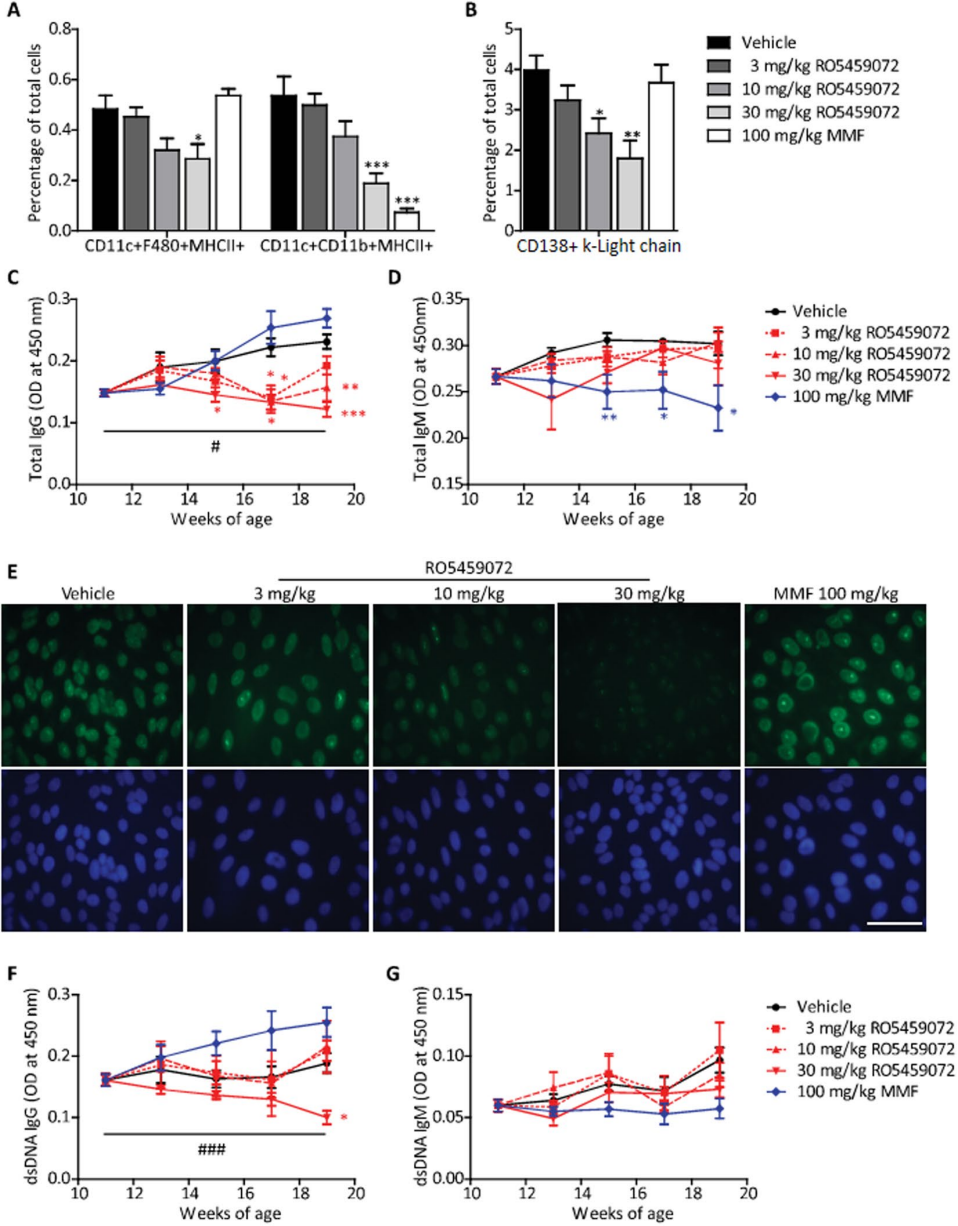
Dose-dependent effects of cathepsin S-inhibition on systemic autoimmunity. From mice of all groups spleen cell suspensions were prepared for flow cytometry using specific antibodies that identify activated (MHC+) antigen-presenting cells, i.e. dendritic cells, macrophages, B cells (**A**) and plasma cells (**B**). Plasma levels of total IgG (**C**) and IgM (**D**) were determined by ELISA at 14 days intervals starting from week 11. (**E**) ANA staining patterns on Hep2 human epithelial cells for plasma, derived from mice of all groups at the end of the study at 1:200 dilution. The respective nuclear DAPI staining is shown in blue colour below (scale 25 µm). Plasma levels of anti-dsDNA IgG (**F**) and IgM (**G**) antibodies were also determined by ELISA. Data are expressed as means ± SEM (n = 8 to 10 in each treatment group). *p < 0.05, **p < 0.01, ***p < 0.001 versus vehicle group. ^#^p < 0.05, ^###^p < 0.001 base line values versus week 19 values.

To compare the potency of Cat-S inhibition with RO5459072 and MMF to suppress autoimmune disease we treated 11 week old nephritic MRL-(Fas)lpr mice with either drug or vehicle for 8 weeks. Both drugs suppressed CD11c+/CD11b+/MHCII+ antigen-presenting cells in spleens of female MRL-(Fas)lpr mice but only RO5459072 suppressed CD11c+/F4/80+/MHCII+ cells (Fig. 1A). As a consequence, Cat-S inhibition (but not MMF) dose-dependently suppressed spleen plasma cells (FACS gating strategies for different spleen cells were illustrated in Supplementary Figure 2) and plasma IgG levels and IgG isotypes (Fig. 1B,C and Supplementary Figure 3). The highest dose of RO5459072 suppressed plasma levels of antinuclear antibodies, whereby the homogenous nuclear staining was indicative of dsDNA antibodies, which was further confirmed by ELISA (Fig. 1E,F). The highest dose of RO5459072 was particularly potent to suppress plasma dsDNA IgG levels below baseline (Fig. 1F). Consistent with its role in MHC-II antigen presentation-dependent immunoglobulin class switch, Cat-S inhibition did not affect total levels of IgM or dsDNA IgM (Fig. 1D and G). In contrast, MMF suppressed plasma IgM levels below baseline (Fig. 1D). Thus, therapeutic Cat-S inhibition suppresses antigen-presenting cells and downstream systemic autoimmunity in MRL-(Fas)lpr mice. MMF does not reduce IgG autoantibodies but suppresses plasma IgM in MRL-(Fas)lpr mice.

### Cathepsin S inhibition versus MMF in an animal model of lupus nephritis

**Figure 2:**
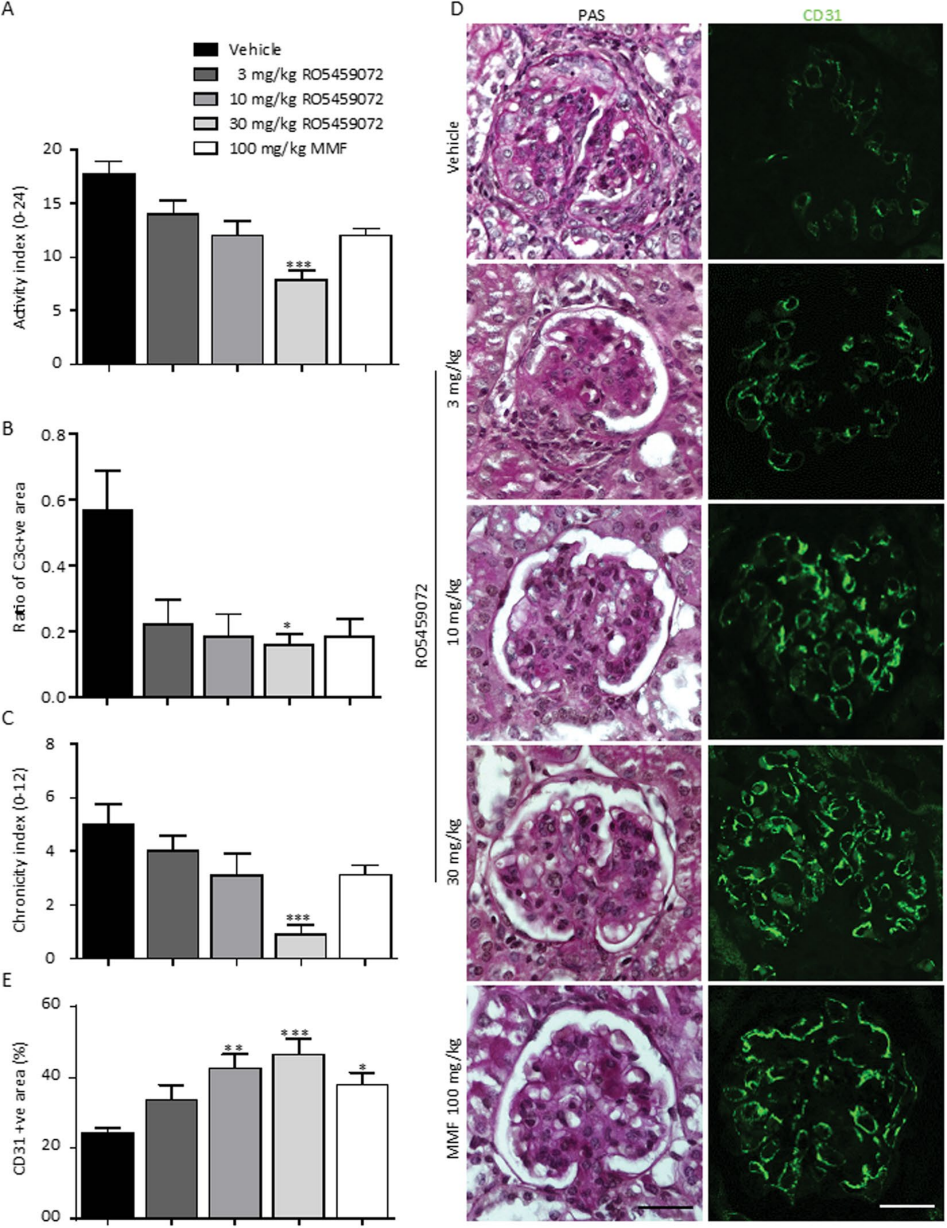
Cathepsin S inhibition is more potent than MMF in suppressing kidney pathology. (**A**) Kidney sections of 19 week old MRL-(Fas)lpr mice of all groups were stained with periodic acid-schiff (PAS). The lupus nephritis disease activity index (**A**, score ranging from 0 to 24), and the lupus nephritis chronicity index score (**C**, ranging from 0 to 12) were determined as markers of kidney damage in lupus nephritis. Representative PAS staining kidney sections and immunofluorescence stainings for the endothelial cell marker CD31 were shown at an original magnification of x400 (scale 25 µm) (**D**). The quantitative analysis of glomerular complement C3 positivity (**B**) and CD31 positivity (**E**) are shown. Data are expressed as means ± SEM. *p < 0.05, **p < 0.01, ***p < 0.001 versus vehicle group.

**Figure 3:**
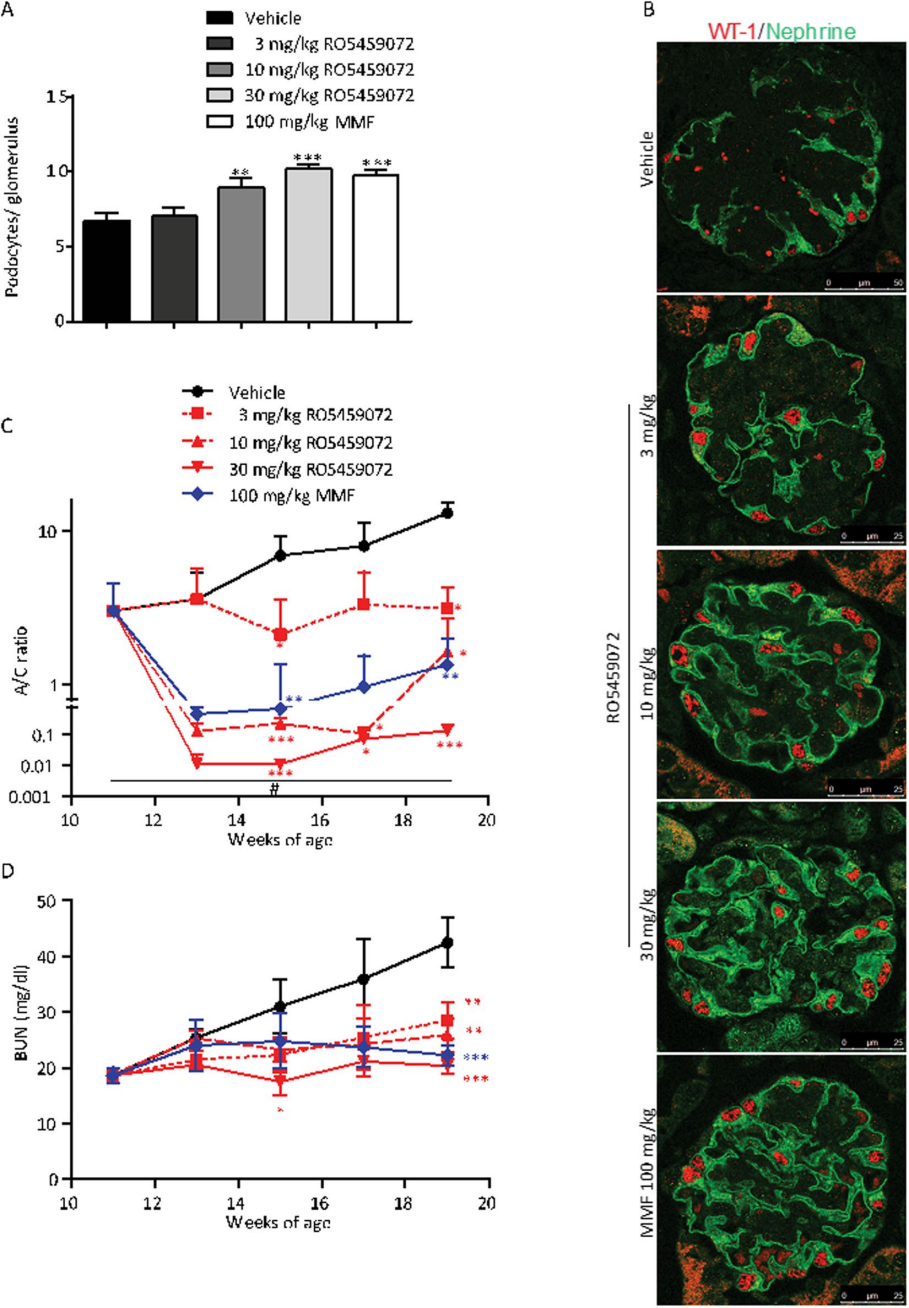
Cathepsin S inhibition has a rapid potent effect on albuminuria. Kidney sections of 19 week old MRL-(Fas)lpr mice of all groups were stained for WT1 and nephrin to quantify podocytes per glomerular cross section (**A**). Representative images are shown in (**B**) at an original magnification of x400 (scale 25 µm). Urinary albumin/creatinine (**A/C**) ratio was determined every 2 weeks and is expressed at logarithmic scale (**C**). Note that the highest dose of RO5459072 reduced A/C ratio quickly at 2 log scales more than MMF. Blood urea nitrogen (BUN) levels were determined at the same time points. Note the linear increae of BUN with time in vehicle-treated mice, while all treatments significantly reduced BUN (**D**). Data are expressed as means ± SEM (n = 8 to 10 in each treatment group). *p < 0.05, **p < 0.01, ***p < 0.001 versus vehicle group. ^#^p < 0.05 base line values versus week 19 values.

To assess the impact of Cat-S inhibition and MMF treatment on autoimmune tissue injury we next examined lupus nephritis-like immune complex disease in MRL-(Fas)lpr mice. RO5459072 dose-dependently reduced the activity and chronicity scores of nephritis, with the highest dose being even more efficacious than MMF therapy (Fig. 2). This was illustrated by less complement C3 deposits, diffuse proliferative glomerular lesions and glomerulosclerosis (Fig. 2A–D left and Supplementary Figure 4). Consistently, both drugs improved the integrity of the glomerular vasculature as illustrated by CD31 staining with the highest dose of RO5459072 demonstrating greater protection (Fig. 2D right-E). This finding was associated with a protective effect also on podocyte number, and proteinuria, the latter being particularly evident at the highest dose of RO5459072 (Fig. 3A–C). It is of note that, Cat-S inhibition had a remarkable early effect on proteinuria as compared to MMF (Fig. 3C). Blood urea nitrogen (BUN) was used as a marker of renal excretory function. All drugs prevented the progressive increase over time seen with vehicle treatment (Fig. 3D). In conclusion, high dose Cat-S inhibition is more potent than MMF to rapidly control lupus nephritis in MRL-(Fas)lpr mice.

### Cathepsin S inhibition and MMF suppress intrarenal inflammation

In lupus nephritis, intrarenal inflammation is a downstream event of systemic autoimmunity and immune complex disease^22^. Both drugs suppressed intrarenal mRNA expression of IL-6, MCP-1/CCL2, RANTES/CCL5, and CXCL2, while that of TNF-α, IFN-γ, and SDF-1/CXCL12 remained unaffected (Supplementary Figure 5A and B). A similar consistent effect was noted on intrarenal expression of the vascular adhesion molecules I-CAM and V-CAM (Supplementary Figure 5C). Flow cytometry of kidneys from mice of all groups revealed that RO5459072 suppressed CD11c+/F4/80+/MHCII+, CD11c+/CD11b+/Ly6c+ and CD11b+/CD103+/CD86+ mononuclear phagocytes and CD45+/CD3+/CD8+, CD45+/CD3+/CD4+ and CD3+/CD4−/CD8− T cells (Supplementary Figure 6, gating strategies were illustrated in Supplementary Figure 7). The impaired lymphocyte recruitment to the kidney was also obvious from CD3 immunostaining (Supplementary Figure 4B and C right). The effect was comparable to that of MMF for most cell populations. Thus, Cat-S and MMF both suppress intrarenal inflammation.

### Late onset of Cat-S inhibition still elicits therapeutic effects in experimental lupus nephritis

To further assess the potency of Cat-S inhibition to control advanced lupus nephritis we performed identical experiments with the highest dose of RO5459072 and MMF, which were not initiated before week 15 of age and continued for only 4 weeks and terminated again at week 19. Very late treatment with RO5459072 also significantly suppressed plasma IgG and its isotypes and anti-dsDNA IgG below baseline, while MMF had no effect (Supplementary Figure 8A–D). MMF treatment showed a trend toward lower plasma IgM levels and did not affect anti-dsDNA IgM as seen upon early treatment (Supplementary Figure 8E and F). RO5459072 and MMF both significantly reduced proteinuria and BUN at week 19 compared to control mice (Supplementary Figure 9A,B). However, very late treatment had a limited effect on the histopathological parameters of lupus nephritis. Only RO5459072 significantly reduced the disease activity index and both drugs failed to significantly suppress the disease chronicity index (Supplementary Figure 9C–E).

### Cathepsin S induces peripheral pathomechanisms on glomerular endothelial cells via protease-activated receptor-2 *in vitro* and in MRL-(Fas)lpr mice

**Figure 4:**
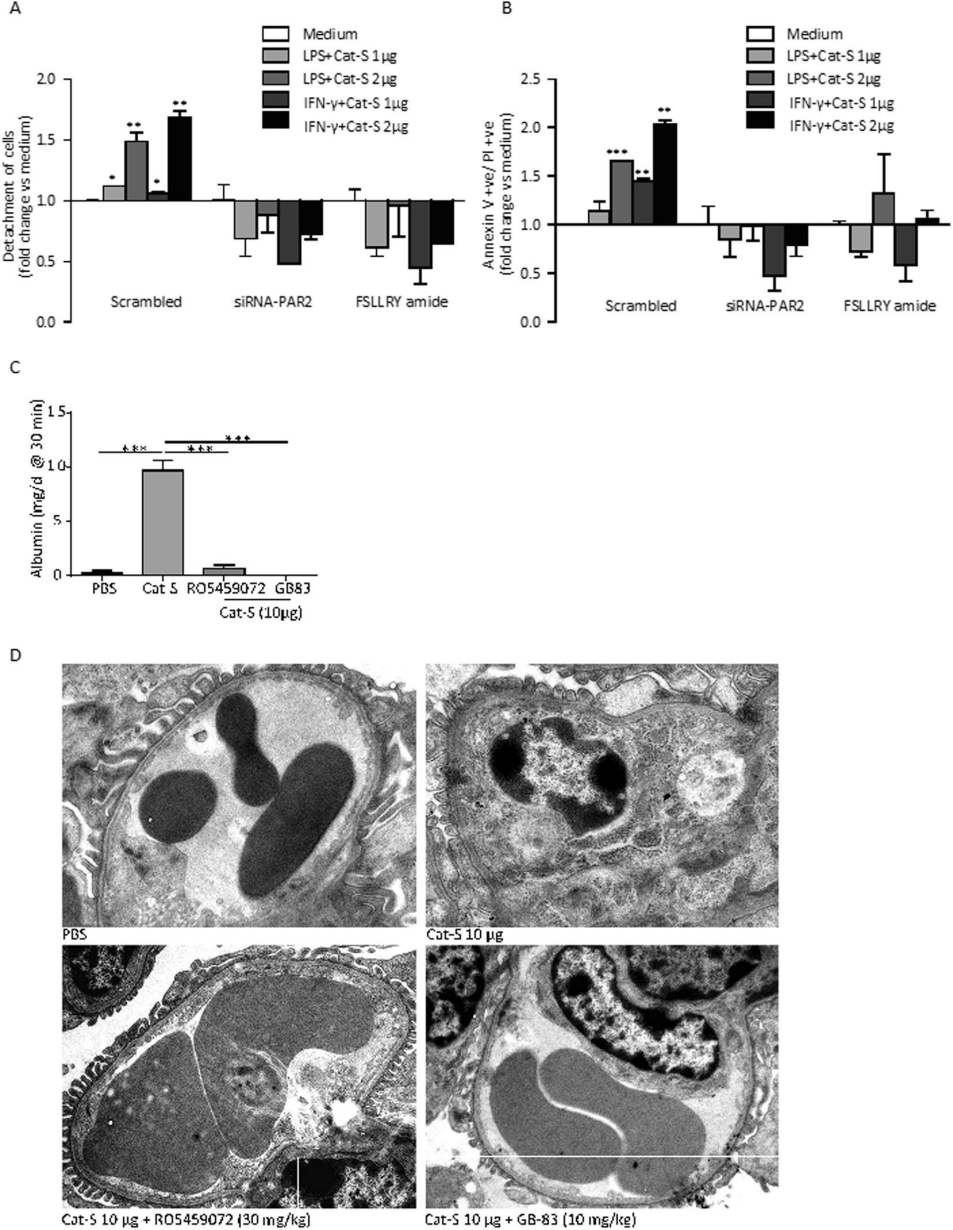
Cathepsin S activates glomerular endothelial cells via protease-activated receptor(PAR)-2. Mouse glomerular endothelial cells were transiently transfected with control siRNA (scrambled) or siRNA specific for PAR-2. Endothelial cell activation with lipopolysaccharide (LPS) or interferon-gamma (IFN-γ) plus different concentrations of Cat-S induced detachment of the cells from the culture dish (**A**). Flow cytometry of the detached cells identified them to be annexin V/propidium iodide (PI) positive (**B**). Cells transfected with PAR-2 siRNA or the PAR-2 inhibitory peptide FSLLRY amide lacked this effect. Female MRL-(Fas)lpr mice were intravenously injected with 10 µg of recombinant Cat-S and albuminuria was determined 30 min later (**C**). Note that the Cat-S-induced albuminuria was entirely blocked by the Cat-S inhibitor RO5459072 or the PAR-2 inhibiting peptide GB83. After 24 h kidneys were harvested for an ultrastructural analysis of glomerular capillaries. Representative images are shown in (**D**). Note that Cat-S injection induced a massive swelling of the glomerular endothelial cells, with loss of the endothelial fenestrations and obliteration of the vascular lumen. Focal podocyte foot process effacement was also present. In contrast RO5459074 and GB83 completely prevented these abnormalities (scale 2 µm). Data in (**C**) are expressed as means ± SEM (n = 3 in each group). ***p < 0.001 versus PBS group.

Cat-S is known to have extracellular protease activity, e.g. by activating PAR-2 on neuronal cells^18, 20^. To test for a similar function on renal endothelial cells we used recombinant Cat-S to stimulate primary murine glomerular endothelial cells (GEnC) and neutralized PAR-2 either with PAR-2-specific siRNA or a PAR-2 antagonist. A combination of Cat-S with LPS or interferon-γ induced detachment of annexin V/PI+ GEnC from the culture dish, which was entirely prevented by either PAR-2-specific siRNA or the PAR-2 antagonist FSLLRY amide (Fig. 4A and B). The beneficial effects of treatments were futher confirmed by flow cytometry using annexin V and PI staining (Fig. 4B). To test, if this cytotoxic mechanism also operates in MRL-(Fas)lpr mice, we intravenously injected 12 week old female MRL-(Fas)lpr mice with recombinant Cat-S and assessed albuminuria and the glomerular ultrastructure by transmission electron microscopy (TEM). Cat-S induced albuminuria within 30 minutes after injection (Fig. 4C), which was associated with a diffuse swelling of the cytoplasm of the endothelial cells within glomerular capillaries leading to partial obstruction of the capillary lumen at 24 h (Fig. 4D). Endothelial cells lost their typical fenestrated appearance, but podocyte ultrastructure was preserved (Fig. 4D). The Cat-S inhibitor RO5459072 as well as the PAR-2 inhibitor GB83 completely prevented albuminuria and endothelial cell injury (Fig. 4C,D). In summary, Cat-S induces specifically endothelial cell injury via PAR-2 *in vitro* and *in vivo*.

### Human neutrophils and monocytes release cathepsin-S upon stimulation

**Figure 5:**
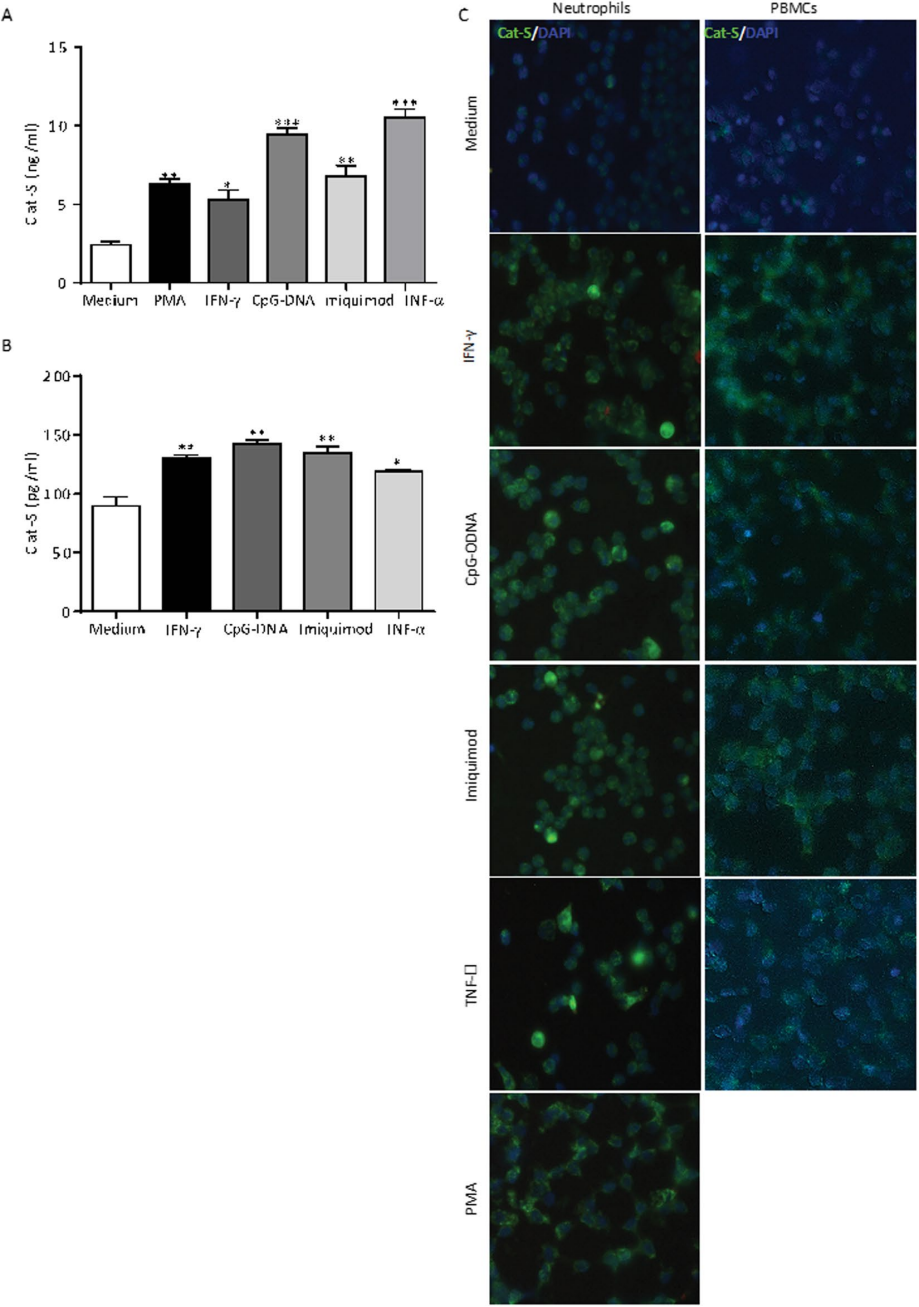
Activated neutrophils and monocytes secrete cathepsin S. Neutrophils (**A**) and PBMCs (**B**) were isolated from human blood and exposed to phorbol myristate acetate (PMA), IFN-γ, the TLR9 ligand CpG-DNA, the TLR7 ligand imiquimod, and TNF-α as indicated. Two hours later Cat-S secretion was quantified in the supernatants by ELISA. The cells were also stained with a FITC-labeled Cat-S antibody (green signal) and DAPI (blue signal) (**C**) (scale 25 µm). Note that all stimuli induce Cat-S expression and secretion in both cell types. Data are means ± SEM from 3 independent experiments. *p < 0.05, **p < 0.01, ***p < 0.001 versus media control.

What is the source of extracellular Cat-S? To address this question we stimulated isolated human neutrophils *in-vitro*, using different ligands for TLR7 agonist, TLR9 agonist, IFN-γ, TNF-α and PMA. After 3 h of incubation, cell culture supernatents were analysed for Cat-S levels by ELISA. All reagents significantly increased the release of Cat-S (Fig. 5A and C). Similar results were obtained with human PBMCs (Fig. 5B,C). Thus, activated neutrophils and monocytes are a source of extracellular Cat-S.

### Cathepsin-S plasma levels in patients with systemic lupus erythematosus and intrarenal expression in lupus nephritis

**Figure 6:**
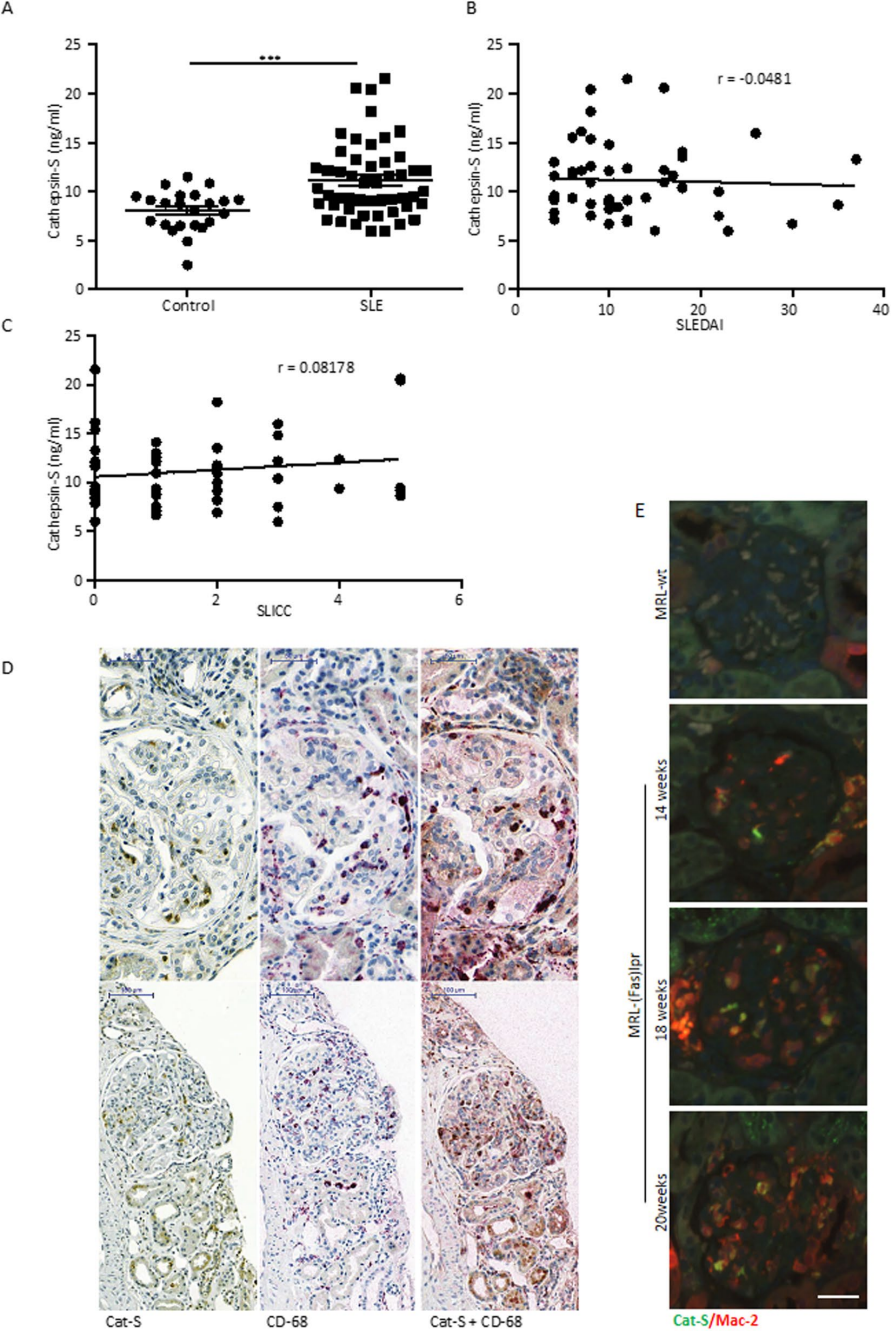
Cathepsin S in human SLE and lupus nephritis. (**A**) Cat-S plasma levels were determined in patients with systemic lupus erythematosus (SLE) and healthy controls by ELISA. The patient characteristics are listed in Table 1. ***p < 0.001 versus healthy controls. (**B**,**C**) In SLE patients Cat-S plasma levels were not correlated with the respective SLE disease activity index (SLEDAI). (**D**) Dual immunostaining for Cat-S and the macrophage marker CD68 of human kidney biopsies showed double positivity in single cells within the glomerular tuft. Cat-S positivity was also noted in CD68 negative tubular epithelial cells. Representative images of two different renal biopsies (class IV lupus nephritis) are shown at the indicated scale 100 µm. (**E**) Dual fluoresence staining for Mac-2 and Cat-S in kidneys of MRL-(Fas)lpr mice of different age groups compared to MRL-wt (scale 25 µm).

To validate these findings in human lupus we first quantified Cat-S levels in plasma samples of patients with SLE. The patient characteristics are shown in Table 1. SLE patients had significantly higher plasma levels of Cat-S compared to healthy controls but there was no correlation of plasma Cat-S levels with SLE disease activity (Fig. 6A–C). Cat-S was also significantly increased in ISN-RPS classification based positive lupus nephritis patients and CKD patients compared to healthy controls (Supplementary Figure 10A–D). Next we assessed intrarenal expression of Cat-S in lupus nephritis. We used immunostaining for Cat-S and the macrophage marker CD68 on serial sections of kidney biopsies from patients with proliferative lupus nephritis. Within glomeruli Cat-S staining was most prominent in single cells within the glomerular tuft, which co-localized with CD68 positivity (Fig. 6D). Glomeruli that lacked CD68+ cell infiltrates hardly showed any Cat-S positivity (Supplementary Figure 10E). Tubules stained positive for Cat-S in all sections (Fig. 6D and Supplementary Figure 10E), as previously reported also from normal kidneys^23^. Further to confirm the association of increased Cat-S levels in MRL-(Fas)lpr mice, we stained kidney sections of MRL-(Fas)lpr mice of different age groups for Cat-S. Increased expression of Cat-S levels in kidneys affected with lupus nephritis were found specifically co-localized to macrophages positive for Mac-2 marker, whereas kidneys of MRL-wt mice were negative for both Mac-2 and Cat-S (Fig. 6E). In conclusion, both mouse and human SLE is associated with elevated levels of Cat-S and in lupus nephritis infiltrating macrophages are a source of intrarenal Cat-S expression.

**Table 1:** Clinical characteristics of SLE patients and healthy control subjects.

	Control n = 23	SLE n = 50
Female, n (%)	17 (74)	41 (82)
Age (years)	42.3 ± 5.6	45.8 ± 1.8
SLEDAI score >4, n (%)	—	50 (100)
SLICC/ACR damage index >1, n (%)	—	33 (66)
Leukopenia, n (%)	—	14 (28)
Thrombocytopenia, n (%)	—	14 (28)
Fatigue, n (%)	—	39 (78)
Arthralgia, n (%)	—	45 (90)
Jaccoud’s arthropathy, n (%)	—	20 (40)
Malar rash, n (%)	—	34 (68)
Pericarditis, n (%)	—	13 (26)
Pleuritis, n (%)	—	14 (28)
Venous thrombosis, n (%)	—	10 (20)
Lupus nephritis, n (%)	—	24 (48)
ISN/RPS class I, n (% of all LN)	—	1 (4)
ISN/RPS class II, n (% of all LN)	—	4 (17)
ISN/RPS class III, n (% of all LN)	—	5 (21)
ISN/RPS class IV, n (% of all LN)	—	14 (58)
CKD, n (%)	—	28 (56)
CKD 1, n (% of all CKD)	—	19 (68)
CKD 2, n (% of all CKD)	—	1 (4)
CKD 3, n (% of all CKD)	—	5 (17)
CKD 4, n (% of all CKD)	—	2 (7)
CKD 5, n (% of all CKD)	—	1 (4)
Hemolytic anemia, n (%)	—	10 (20)
Antinuclear antibodies, n (%)	—	47 (94)
Anti-dsDNA IgG, n (%)	—	31 (62)

SLEDAI: Systemic lupus erythematosus activity index, SLICC/ACR: Systemic Lupus International Collaborating Clinics/American College of Rheumatology, CKD: Chronic kidney disease.

## Discussion

The essential roles of Cat-S in MHC-II processing and in PAR-2-mediated vascular injury prompted us to speculate that therapeutic Cat-S inhibition could elicit a dual effect on autoimmune vascular disease that is more selective and superior to conventional immunosuppressive drugs. Our data validate this concept for RO5459072-mediated Cat-S inhibition in lupus nephritis of MRL-(Fas)lpr mice. In addition, selective Cat-S inhibition avoids the unspecific immunosuppressive effect of MMF on plasma IgM that are important for first line host defense. However, Cat-S inhibition will still impair MHC-II-driven IgG responses needed for host defense. As such, some effect on host defense can also not be avoided with Cat-S inhibition. We had previously shown that another, closely related Cat-S antagonist, RO5451111, can suppress the priming of autoreactive T and B cells and germinal center formation in MRL-(Fas)lpr mice^17, 23^. The present study further explores this novel therapeutic concept in five different ways:

First, we tested the bioactivity of the additional Cat S inhibitor RO5459072 and found that it elicits a clear dose-dependent immunomodulatory effect on almost each of the analyzed parameters of systemic autoimmune disease and autoimmune tissue injury in MRL-(Fas)lpr mice. This consistent dose-dependency is in support of a specific pharmacological inhibition of the Cat-S-dependent pathophysiology in systemic autoimmunity.

Second, we compared the therapeutic efficacy of the Cat-S inhibitor RO5459072 with MMF at a dose proven to efficiently control SLE and lupus nephritis of MRL-(Fas)lpr mice^24–26^. MMF potently suppressed all aspects of SLE and lupus nephritis in MRL-(Fas)lpr mice, although a relatively poor effect on dsDNA IgG levels was noted. We found Cat-S inhibition with 30 mg/kg RO5459072 from week 11–19 to be more potent than MMF in controlling spleen macrophage activation, plasma cell expansion, hypergammaglobulinemia, and anti-dsDNA-IgG production as well as the activity and chronicity indices of lupus nephritis. Most importantly, unlike MMF Cat-S inhibition did not suppress plasma IgM, which are an important component for the immediate innate host defense against pathogens^27^. This selective immunosuppressive effect sparing IgM levels might translate into a lower risk for treatment-related infections that is still a problem with currently used therapeutic regimen for autoimmune diseases^2^.

Third, we compared delayed versus very late onset of treatment for both drugs in order to evaluate the therapeutic window in autoimmune disease. Very late onset of 30 mg/kg RO5459072 was still more potent than MMF to effectively suppress hypergammaglobulinemia and anti-dsDNA production, albuminuria, and lupus nephritis disease activity. However, the capacity of both drugs to suppress lupus nephritis chronicity was much lower than with the earlier start of the treatment. This finding is in line with the general concept of “the earlier-the better” that applies for autoimmune and non-autoimmune forms of progressive CKD^28^. Indeed, at more advanced stages of the disease process the vasoprotective effect of Cat-S inhibition may be less pronounced than at earlier stages.

Fourth, we explored the capacity of RO5459072 to block Cat-S-mediated PAR-2 activation and injury of endothelial cells *in vitro* and in MRL-(Fas)lpr mice. PAR-2 suppression specifically impaired the Cat-S-driven alteration of glomerular endothelial cells that were preactivated with the TLR4 agonist LPS or IFN-γ. Cat-S or PAR-2 inhibition both prevented Cat-S-induced injury to the glomerular filtration barrier, i.e. endothelial cell injury and barrier dysfunction (albuminuria). These data are consistent with our recent findings in diabetic mice with Cat-S-mediated endothelial dysfunction^19^ and are in line with other reports on Cat-S-specific PAR-2 activation^18, 20^.

Finally, we assessed the expression of Cat-S in human SLE and lupus nephritis and identified activated neutrophils and macrophages as a source of circulating Cat-S. While neutrophils contribute to disease activity mostly via NET formation outside the kidney^29^, activated macrophages also recruit inside the kidney and contribute to autoimmune tissue injury^30–34^. SLE patients displayed higher plasma levels of Cat-S but independent from disease activity. The same was observed for patients with rheumatoid arthritis^35^, which may relate to a low threshold of Cat-S induction in diseased individuals. However, increased plasma levels of Cat-S have been observed in lupus patients with nervous system disorders^36^ or numerous patient cohorts with other chronic disease entities and were shown to imply higher mortality^37, 38^. This finding may be clinically important because the presence of lupus nephritis is also associated with poor overall survival^39^.

In summary, Cat-S has a dual effect in autoimmune disease. First, Cat-S is a non-redundant element of MHC-II-mediated autoantigen presentation and second, Cat-S secreted, e.g. by activated immune cells, promotes PAR-2-driven endothelial injury in organs affected by autoimmune tissue inflammation. As such initiating Cat-S inhibition even after the onset of clinically apparent autoimmune disease not only specifically suppresses the central immunological pathomechanism shared by all autoimmune diseases^17^, i.e. MHC-II-mediated autoantigen presentation but also protects the peripheral vascular interface and thereby suppresses leukocyte recruitment, tissue inflammation and remodeling of organs affected by autoimmune injury, especially when treatment is initiated early. The highly specific blockade of MHC-II processing with Cat-S inhibition avoids the broad immunosuppressive effect of nonspecific immunosuppressant drugs like MMF that also suppress plasma IgM, which are essential for immediate host defense. We therefore conclude that Cat-S inhibition may represent a next generation immunomodulatory strategy for all types of autoimmune diseases by combining highly specific neutralization of autoantigen-presentation with vasoprotective and anti-inflammatory effects, which can have potent additive effects especially when initiated early during the disease course.

## Materials and Methods

### *In-vitro* charecterization of the cathepsin S inhibitor RO5459072

RO5459072 was provided by F. Hoffmann-La Roche, Ltd., Basel, Switzerland. It is a competitive inhibitor of the active site of Cat-S and its nitrile function allows covalent reversible inhibition of Cat-S. The synthesis of RO5459072 has been described in WO 2010121918 and WO 2013068434. Recombinant cathepsins were produced in-house or were purchased from commercial vendors. Initial enzymatic assays to determine isoform-selectivity *in vitro* were performed as previously described^21, 23^. As the selectivity assays indicated K_i_ values for the interaction of RO5459072 with human Cat-S that were below the concentration of enzyme in the assays suggesting tight binding, we proceeded with a detailed kinetic analysis^40, 49^. For this the Cat-S-dependent hydrolysis of the fluorogenic substrate Z-Phe-Arg-AMC (Sigma Aldrich) was followed at various inhibitor concentrations. The active fraction of Cat-S was detemined as 70% with a HPLC assay measuring modification of Cat-S with the covalent inhibitor LHVS. The buffer (100 mM potassium phosphate, 50 mM NaCl, 5 mM EDTA, 5 mM TCEP, pH 6) was thoroughly degassed prior to perfoming measurements to prevent oxidation of the catalytic cystein residue by dissolved oxygen. For the K_i_-determination the substrate was employed at 10 µM = K_M_. All kinetic measurements were performad on a SFM-3000 stopped-flow instrument (Bio-Logic Science Instruments) and data were analyzed with GraphPad Prism v5. RO5459072 did not show any signals regarding drug safety in extensive screening studies in mice, rats, monkeys and humans that preceeded the currently ongoing clinical trial (NCT01701985).

### Mice and protocol for experimental SLE and lupus nephritis

Four-week-old female MRL-(Fas)lpr mice were purchased from Harlan Winkelmann (Borchen, Germany) and kept under pathogen-free conditions in a 12 hour light and dark cycle with free access to food and water. All animal experiments were performed in accordance with the European protection laws of animal welfare, and with approval by the local government authorities Regierung von Oberbayern (reference number: 55.2.1-54-2532.2-11-12). At 11 or 15 weeks of age only mice with proteinuria (Albustix, Bayer AG, Leverkusen, Germany) were randomized into five different groups (n = 10) receiving different medicated diets: Three groups were fed with chow mixed with either 3, 10 or 30 mg/kg/day RO5459072, another group’s chow was mixed with 100 mg/kg/day mycophenolate mofetil (MMF), and one group received a standard diet (vehicle). The dose of MMF was chosen according to several studies reporting strong efficacy on SLE and lupus nephritis in MRL-(Fas)lpr mice^24–26^. Food intake was measured once weekly and was stable at approximately 5 g food per day throughout the study period with no significant difference of food intake or body weight between any of the groups. At the age of 19 weeks all mice were sacrificed by cervical dislocation. Blood and urine samples were collected at different time points as indicated. In other experiments 7 week old female MRL-(Fas)lpr mice were randomized into three groups (n = 3) to receive a single intravenous injection of 10 µg sterile recombinant Cat-S and one group receiving vehicle (100 µl sterile PBS). One of the Cat-S groups was pretreated with 30 mg/kg/day RO5459072 starting one week prior to Cat-S injection; another group was pretreated with a total of four daily intra-peritoneal injections of the PAR-2 inhibitor GB83 (10 mg/kg).

Plasma samples were collected from mice during the feeding cycle on weeks 2, 4, 6 and 8 on a variable schedule of individuals after initiation of feeding with the drug-food admix on week 1. The levels in food were calculated to achieve doses of 3, 10 and 30 mg/kg/day. EDTA-treated plasma samples (29 per dose level) were frozen and subsequently analysed for the parent drug by HPLC-MS/MS. A column-trapping method (Phenonenex Luna C18 and Thermo Hypersil Gold C8 HPLC columns eluted with formic acid/water/acetonitrile mixtures on a Waters Acquity HPLC system) was used to separate the drug which was quantified using a Sciex API4000 MS/MS. The analytical range was 10–10,000 ng/ml and a stable label internal standard was used. Urine samples were collected from all mice at baseline and after 0.5, 1, 1.5, 2, 4, 6, 12, and 24 h. All experiments were performed according to German animal protection laws and had been approved by the local government authorities.

### Morphological evaluation

Kidney tissue samples were collected from all mice and fixed overnight in 4% buffered formalin. Subsequently the fixed tissue samples were embedded in paraffin and cut into 4 µm thick serial sections. Kidney paraffin sections were stained with periodic acid-Schiff (PAS) reaction using standardized protocols as described^41^. Scoring of lupus nephritis activity and chronicity indices was performed according to human lupus nephritis^42, 43^. Methenamine silver staining was performed on paraffin embedded kidney sections and semiquantitatively scored form 0–3 by a blinded observer. For immunostaining the following primary antibodies were used: anti-mouse CD3 (Abcam, Cambridge, UK), complement C3c (Biobyt Ltd, Cambridge, UK), CD31 (Dianova GmbH, Hamburg, Germany), WT1 (Santa Cruz Biotechnology, Dallas, Texas) and nephrin (Acris Antibodies, San Diego, CA). The immunostaining for C3c and CD31 were analyzed using the ImageJ software by measuring positively stained area and dividing it by the total area of the glomerulus and reported as percentage of +ve area. Sections stained for WT1/nephrin and CD3 were evaluated by counting positive cells per glomerulus. All the morphometric analysis were carried out in a blinded fashion.

### Biomarkers of renal function and autoantibody production

Urinary albumin/creatinine ratios were determined using the Mouse Albumin ELISA Quantitation set (Bethyl Laboratories, Montgomery, TX) and the Creatinine FS kit (DiaSys Diagnostic Systems, Holzheim, Germany)^44^. BUN levels were determined in plasma samples using a Urea FS kit (DiaSys Diagnostic Systems). Mouse IgM, IgG and its isotypes IgG1, IgG2a and IgG2b plasma levels were measured using ELISA Sets (Bethyl Laboratories) at a plasma dilution of 1:1,000 to 1:100,000. Double-stranded DNA IgG and IgM antibodies were quantified as described^45^. Briefly, 96 well Nunc-Immuno plates (Thermo Fisher Scientific, Roskilde, Denmark) were coated with poly-L-lysine (1:1 diluted with PBS) and incubated for two hours. After washing the plates, the plates were coated with 1 µg/ml mouse tail dsDNA sparing the wells for standards, which were coated with anti-mIgG and anti-mIgM (Bethyl Laboratories). The standard curve was fixed from purified mIgG and mIgM (Bethyl Laboratories). The plasma samples were diluted 1:1,000 and added into the wells. After 1 h the assay was developed using HRP-labelled goat anti-mIgG and anti-mIgM and TMB substrate (BD Bioscience, San Diego, CA). Absorbance was measured at 450 nm using a Tecan Microplate Reader (Biotek, Winnoski, VT). Indirect immunofluorescence was used to quantify ANA in mouse plasma. 1:200 diluted mouse plasma was applied to slides covered with HEp-2 cells (Bio-Rad Laboratories, Redmond, WA, USA) as described^43^.

### Transmission electron microscopy

Mouse kidney tissue was fixed in 2% paraformaldehyde/2% glutaraldehyde and 0.1M sodium phosphate buffer (pH 7.4) for 24 hours and processed as described^19^. Samples were analyzed with a 1200EX electron microscope (JEOL, Tokyo, Japan).

### Immunohistochemistry in human renal biopsies

Renal biopsies from patients with active lupus nephritis were selected from the files of the Department of Pathology and Immunology, University Hospital of Geneva. Informed consent was obtained in all cases. The renal tissue was fixed in 4% PBS-buffered formalin and embedded in paraffin. For all biopsy specimens, standard analysis using light microscopy, immunofluorescence (with anti-IgA, G, M, and anti-complement C1q, C3, C4 and C5b-9 antibodies) and electron microscopy was performed. Scoring of lupus nephritis activity and chronicity indices was performed according to the ISN/RPS 2003 classification^46^. For immunostaining, serial paraffin sections were stained with the primary antibodies anti-cathepsin S (polyclonal goat anti-human Cat-S, Abcam, Cambridge, UK) and anti-CD68 (DakoCytomation, Glostrup, Denmark), or double stained with anti-cathepsin S and anti-CD68 as previously reported^19^. 8 renal biopsies (2 class II, 2 class III, 3 class IV and 1 class V) were analyzed.

### Cathepsin-S levels in SLE patients plasma samples

Plasma samples from Lupus patients listed in the Munich cohort of active SLE patients, as well as plasma samples form healthy controls were stored at −20 °C untill all the analyses were completed. Cat-S plasma levels were measured by using a commercially available Cat-S ELISA kit (R&D Systems, Minneapolis, MN, USA). All patients had given written informed consent.

### Cell culture assays

GEnCs were suspended in 1 M nucleofection buffer mixed with siRNA to PAR-2 (Ambion, Cat. AM16708) or scrambled siRNA as control (Cat. 4390843). Nucleofections were performed using a Lonza electroporation instrument and efficacy was measured by qRT-PCR. After transfection the cells were cultured in RPMI medium containing 1% FCS and 1% PS. Another set of GEnCs were prestimulated with the PAR-2 inhibitor (FSLLRY-amide) for 30 min. Once the cells adhered to the plates, they were plasma-fasted for 2 h in fresh incomplete RPMI medium and stimulated for 20 hours with LPS (0.5 µg/ml) or IFN-γ (1000 IU) in the presence of either 1 µg or 2 µg Cat-S, or left as unstimulated media controls. One group of GEnCs were not transfected but were prestimulated with the PAR-2 inhibitor (FSLLRY-amide) for 30 min before stimulation. Detached cells in the supernatant were counted using a scepter hand held automated cell counter (Millipore, Cat. PHCC00000, Billerica, MA, USA) and then stained with Annexin/PI (Miltenyi Biotec GmbH, Bergisch Gladbach, Germany) for flow cytometric analysis.

Human neutrophils were isolated from full blood samples of a healthy donor, who had given written informed consent, using a standard dextran sedimentation method followed by Lymphoprep (Stemcell Technologies, Cologne, Germany) density centrifugation. 0.5 × 10^6^ or 5 × 10^6^ neutrophils were suspended in 200 to 500 µl RPMI media and seeded into 8 well µ slides or 12 well plates (Ibidi, Martinsried, Germany). The cells were stimulated for 3 h with phorbol 12-myristate 13-acetate (25 nM; PMA, Sigma-Aldrich, Steinheim, Germany), IFN-γ (1000 IU/ml), imiquimod (5 µg/ml, Invivogen, San Diego, California), CpG DNA (25 µg/ml, Invivogen) or TNF-α (200 nM, Immunotools, Germany). A Cat-S ELISA (R&D Systems, Minneapolis, MN, USA) was used to quantify protein in the supernatant. Cells on 8 well µ slides were immunostained for Cat-S (Abcam).

For isolating human PBMCs full blood samples were collected from a healthy donor, as described above. The blood was diluted 1:1 with sterile PBS and layered on a Lymphoprep solution (Stemcell Technologies). By subsequent density gradient centrifugation at 800 g for 30 min at room temperature PBMCs were separated from other blood components. The PBMCs were washed with RPMI medium and seeded at a concentration of 0.5 × 10^6^ or 1 × 10^6^ cells/well in 12 well plates or on 8 well µ slides. The cells were stimulated with IFN-γ (1000 IU/ml), imiquimod (5 µg/ml), CpG-DNA (25 µg/ml) or TNF-α (200 nM). After 2 hours the supernatant was collected, Cat-S levels were measured by ELISA (R&D Systems) and the cells on the 8 well µ slides were double stained with FITC-anti-Cat-S (Abcam) and DAPI.

### Pharmacodymamics of Cathepsin S inhibition by RO5459072 in human B-cells and mouse spleens *in vivo*

For the determination of *in-vivo* enzyme inhibition activity of Cat-S by RO5459072 BALB/c mice were used. After oral dosing of 0.1 mg/kg to 100 mg/kg of RO5459072, mice were sacrificed after 7 hours and spleens harvested. Invariant chain was quantified by western blot from mouse splenocyte supernatants loaded onto 10% SDS-PAGE gel. Upon transfer onto a nitrocellulose membrane rabbit anti-CD74 (BD Biosciences) was used.

Human B-cells were isolated from whole blood using the Whole Blood CD19 Microbeads (Cat#130-090-880, Milteny) with the Whole Blood Column Kit (130-093-545, Milteny) according to the instruction leaflet. Isolated B-cells were adjusted to a cell density of 1 × 10^6^ cells/ml in RPMI medium containing 10% FCS and 0.05 mM β-Mercaptoethanol (media and supplements are from Gibco). For each condition 100 µl of cell suspension was transferred into one well of a 96 well cell culture plate, respectively. For detection of p10, cells were treated with different concentrations of RO5459072 for 16 h in a cell culture incubator (37 °C; 5% CO_2_). Afterwards cells were lysed by adding 100 µl 2x NP-40 lysis buffer (20 mM Tris pH 8.0; 137 mM NaCl; 10% glycerol; 1% NP-40; 2 mM EDTA) containing HALT Protease inhibitor (Cat#78429, Pierce) for 30 min on ice. Before detection of accumulated p10-MHC-II complexes, interfering Ii-MHC-II associates were removed from the cell lysates. Therefore the cell lysates were incubated twice for 2 h at 4 °C on two different ELISA plates (Maxisorp/NUNC) which were previously coated with CD74–18/3 (5 µg/ml/overnight) and subsequently blocked with blocking buffer (50 mM Tris, 140 mM NaCl, 5 mM EDTA, 0.05% NP40 and 0.25% gelantine pH 7.4) for 1 h at 37 °C. The CD74-18/3 antibody detects uncleaved invariant chain MHC-II complexes only. Afterwards p10-MHC-II complexes were analyzed by ELISA. For this purpose black Maxisorp plates from NUNC have been coated o/n with PIN.1 (5 µg/ml) from abcam (Cat#ab22603). The next day plates were blocked with blocking buffer for 1 h at 37 °C. Lysates containing 25,000 B-cells per condition were transferred to each well of the ELISA plate respectively and incubated together with the secondary antibody CD74-3/5-Biotin (15 ng/ml) o/n at 4 °C on a shaker. For signal amplification samples were incubated for 30 min with High Sensitive Streptavidin-HRP, 50 ng/ml (Cat#21134, Pierce). The amount of p10 was detected after 30 min of incubation with the QuantaBluTM Fluorogenic Substrate (Cat#15162, Pierce) at 325 nm excitation and 420 nm emission. In between each procedure of the ELISA assay the plates were washed twice with PBS−/− containing 0.05% TWEEN-20.

### Flow cytometry

Whole kidneys and tissue from 1/3 of a spleen were processed to single cell suspensions and stained with the following antibodies purchased from BD Bioscience (San Diego, CA, USA): anti-mouse CD3e-FITC, Ly6c-FITC, CD11b-PE, CD11c-PE, CD45-PE, k Light Chain-PE, CD8a-PerCP, CD4-APC, CD11b-APC, CD138-APC, CD86-FITC. Also used for staining were anti-mouse MHC-II-FITC and CD103-APC (both from eBioscience, San Diego, CA) and F4/80-APC from Bio-Rad (Raleigh, NC, USA). Detached GEnCs were stained with annexin/PI (Miltenyi Biotec, Germany). The FACSCalibur flow cytometer was used for acquiring the cells and analysis was done using the CellQuest software.

### Quantitative Real-time PCR

Total RNA was isolated from kidney tissue using PureLink RNA Mini Kit (Invitrogen, Calsbad, CA). The RNA’s quality and concentration was measured with a NanoDrop (Thermo Scientific, NanoDrop products, Wilmington, DE) and later cDNA was synthesized using SuperScript II Reverse Transcriptase (Invitrogen). Gene expression rates were determined by quantitative real-time PCR applying the SYBR Green Dye detection system on the LightCycler 480 (Roche Diagnostics, Mannheim, Germany). The following primers were purchased from Metabion, Martinsried, Germany: Ccl2 (R: ATTGGGATCATCTTGCTGGT and F: CCTGCTGTTCACAGTTGCC), Ccl5 (R: GTGCCCACGTCAAGGAGTAT and F: CCACTTCTTCTCTGGGTTGG), Cxcl2 (R: TCCAGGTCAGTTAGCCTTGC and F: CGGTCAAAAAGTTTGCCTTG), Il6 (R: ACCAGAGGAAATTTTCAATAGGC and F: TGATGCACTTGCAGAAAACA), Ifn-γ (R: TGAGCTCATTGAATGCTTGG and F: ACAGCAAGGCGAAAAAGGAT), Tnf-α (R: TAGACAAGGTACAACCCATCGG and F: AGCCTCTTCTCATTCCTGCT), Vcam-1 (R: ACTTGTGCAGCCACCTGAGATC and F: GCTATGAGGATGGAAGACTCTGG), Icam-1 (R: AACAGTTCACCTGCACGGAC and F: GTCACCGTTGTGATCCCTG), SDF-1 (R: TTTCAGATGCTTGACGTTGG and F: GCGCTCTGCATCAGTGAC). For every gene RT- and water samples were used as negative controls as well as 18S rRNA acting as an endogenous control^47, 48^. For analysis the comparative threshold cycle (ΔΔCp) method was used.

### Statistical analysis

All the data were first checked for Gaussian distribution using Kolmogorov-Smirnov test. Normally distributed data were analyzed by ordinary one-way ANOVA followed by Dunnett’s multiple comparisons as post hoc test, while data not showing a Gaussian distribution were analysed by nonparametric ANOVA (Kruskal-Wallis test) followed by Dunn’s multiple comparisons as post hoc test. Comparisons of endpoint time-points to baseline time-points were performed using a Student t-test. P < 0.05 was considered statistically significant. Human samples were analysed for the Gaussian distribution applying D’Agostino-Pearson test and Cathepsin-S levels between healthy and SLE samples were analysed using nonparametric t-test. Correlation co-efficient “r” was calculated using Spearman nonparametric correlation for the data set of Cathepsin-S levels vs SLEDAI or SLICC. Statistics were calculated using the software GraphPad Prism (version 5; GraphPad, San Diego, CA, USA).

## Electronic supplementary material

Supplementary information

## References

[1] Goodnow CC (2007). Multistep pathogenesis of autoimmune disease. Cell.

[2] Hogan J, Avasare R, Radhakrishnan J (2014). Is newer safer? Adverse events associated with first-line therapies for ANCA-associated vasculitis and lupus nephritis. Clinical journal of the American Society of Nephrology: CJASN.

[3] Aringer M (2009). Adverse events and efficacy of TNF-alpha blockade with infliximab in patients with systemic lupus erythematosus: long-term follow-up of 13 patients. Rheumatology.

[4] Merrill JT (2010). Efficacy and safety of rituximab in moderately-to-severely active systemic lupus erythematosus: the randomized, double-blind, phase II/III systemic lupus erythematosus evaluation of rituximab trial. Arthritis and rheumatism.

[5] Roche PA, Furuta K (2015). The ins and outs of MHC class II-mediated antigen processing and presentation. Nature reviews. Immunology.

[6] Gupta S, Singh RK, Dastidar S, Ray A (2008). Cysteine cathepsin S as an immunomodulatory target: present and future trends. Expert opinion on therapeutic targets.

[7] Blum JS, Cresswell P (1988). Role for intracellular proteases in the processing and transport of class II HLA antigens. Proceedings of the National Academy of Sciences.

[8] Driessen C (1999). Cathepsin S controls the trafficking and maturation of MHC class II molecules in dendritic cells. The Journal of cell biology.

[9] Germain RN (1994). MHC-dependent antigen processing and peptide presentation: Providing ligands for T lymphocyte activation. Cell.

[10] Riese RJ (1998). Cathepsin S activity regulates antigen presentation and immunity. The Journal of clinical investigation.

[11] Riese RJ (1996). Essential role for cathepsin S in MHC class II-associated invariant chain processing and peptide loading. Immunity.

[12] Roche PA, Marks MS, Cresswell PJ (1991). Formation of a nine-subunit complex by HLA class II glycoproteins and the invariant chain. Nature.

[13] Shi GP, Munger JS, Meara JP, Rich DH, Chapman HA (1992). Molecular cloning and expression of human alveolar macrophage cathepsin S, an elastinolytic cysteine protease. The Journal of biological chemistry.

[14] Stoeckle C (2012). Cathepsin S dominates autoantigen processing in human thymic dendritic cells. Journal of autoimmunity.

[15] Baugh M (2011). Therapeutic dosing of an orally active, selective cathepsin S inhibitor suppresses disease in models of autoimmunity. Journal of autoimmunity.

[16] Saegusa K (2002). Cathepsin S inhibitor prevents autoantigen presentation and autoimmunity. The Journal of clinical investigation.

[17] Bernard NJ (2014). Connective tissue diseases. Inhibiting cathepsin S to treat SLE and lupus nephritis. Nature reviews. Rheumatology.

[18] Elmariah SB, Reddy VB, Lerner EA (2014). Cathepsin S signals via PAR2 and generates a novel tethered ligand receptor agonist. PloS one.

[19] Kumar SV (2015). Neutrophil Extracellular Trap-Related Extracellular Histones Cause Vascular Necrosis in Severe GN. Journal of the American Society of Nephrology: JASN.

[20] Zhao P (2014). Cathepsin S causes inflammatory pain via biased agonism of PAR2 and TRPV4. The Journal of biological chemistry.

[21] Kumar Vr S (2016). Cathepsin S Cleavage of Protease-Activated Receptor-2 on Endothelial Cells Promotes Microvascular Diabetes Complications. Journal of the American Society of Nephrology: JASN.

[22] Lech M (2011). IRF4 Deficiency Abrogates Lupus Nephritis Despite Enhancing Systemic Cytokine Production. Journal of the American Society of Nephrology.

[23] Rupanagudi KV (2015). Cathepsin S inhibition suppresses systemic lupus erythematosus and lupus nephritis because cathepsin S is essential for MHC class II-mediated CD4 T cell and B cell priming. Annals of the rheumatic diseases.

[24] Jonsson CA, Svensson L, Carlsten H (1999). Beneficial effect of the inosine monophosphate dehydrogenase inhibitor mycophenolate mofetil on survival and severity of glomerulonephritis in systemic lupus erythematosus (SLE)-prone MRLlpr/lpr mice. Clinical and experimental immunology.

[25] Lui SL (2002). Effect of mycophenolate mofetil on severity of nephritis and nitric oxide production in lupus-prone MRL/lpr mice. Lupus.

[26] Van Bruggen MC, Walgreen B, Rijke TP, Berden JH (1998). Attenuation of murine lupus nephritis by mycophenolate mofetil. Journal of the American Society of Nephrology: JASN.

[27] Boes M (2000). Role of natural and immune IgM antibodies in immune responses. Molecular immunology.

[28] Gross O (2012). Early angiotensin-converting enzyme inhibition in Alport syndrome delays renal failure and improves life expectancy. Kidney international.

[29] Bosch X (2011). Systemic lupus erythematosus and the neutrophil. The New England journal of medicine.

[30] Bethunaickan R (2011). A unique hybrid renal mononuclear phagocyte activation phenotype in murine systemic lupus erythematosus nephritis. Journal of immunology.

[31] Allam R, Anders HJ (2008). The role of innate immunity in autoimmune tissue injury. Current opinion in rheumatology.

[32] Anders HJ (2004). Late onset of treatment with a chemokine receptor CCR1 antagonist prevents progression of lupus nephritis in MRL-Fas(lpr) mice. Journal of the American Society of Nephrology: JASN.

[33] Kurts C, Panzer U, Anders HJ, Rees AJ (2013). The immune system and kidney disease: basic concepts and clinical implications. Nature reviews. Immunology.

[34] Vielhauer V, Kulkarni O, Reichel CA, Anders HJ (2010). Targeting the recruitment of monocytes and macrophages in renal disease. Seminars in nephrology.

[35] Ruge T, Sodergren A, Wallberg-Jonsson S, Larsson A, Arnlov J (2014). Circulating plasma levels of cathepsin S and L are not associated with disease severity in patients with rheumatoid arthritis. Scandinavian journal of rheumatology.

[36] Zhang TP (2016). Plasma levels of adipokines in systemic lupus erythematosus patients. Cytokine.

[37] Arnlov J (2012). Cathepsin S as a biomarker: where are we now and what are the future challenges?. Biomarkers in medicine.

[38] Jobs E (2011). Association between serum cathepsin S and mortality in older adults. Jama.

[39] Manger K (2002). Definition of risk factors for death, end stage renal disease, and thromboembolic events in a monocentric cohort of 338 patients with systemic lupus erythematosus. Annals of the rheumatic diseases.

[40] Copeland, R. A. *Tight binding inhibitors*. 2nd edn, 305–317 (Wiley-VCH, Inc., 2000).

[41] Patole PS (2007). Coactivation of Toll-like receptor-3 and -7 in immune complex glomerulonephritis. Journal of autoimmunity.

[42] Austin HA, Muenz LR, Joyce KM, Antonovych TT, Balow JE (1984). Diffuse proliferative lupus nephritis: identification of specific pathologic features affecting renal outcome. Kidney international.

[43] Allam R (2008). Viral 5′-triphosphate RNA and non-CpG DNA aggravate autoimmunity and lupus nephritis via distinct TLR-independent immune responses. European journal of immunology.

[44] Pawar RD (2009). Bacterial lipopeptide triggers massive albuminuria in murine lupus nephritis by activating Toll-like receptor 2 at the glomerular filtration barrier. Immunology.

[45] Kulkarni O (2007). Spiegelmer Inhibition of CCL2/MCP-1 Ameliorates Lupus Nephritis in MRL-(Fas)lpr Mice. Journal of the American Society of Nephrology.

[46] Weening JJ (2004). The classification of glomerulonephritis in systemic lupus erythematosus revisited. Journal of the American Society of Nephrology: JASN.

[47] Lech M, Anders HJ (2014). Expression profiling by real-time quantitative polymerase chain reaction (RT-qPCR). Methods in molecular biology.

[48] Lech M (2012). Quantitative expression of C-type lectin receptors in humans and mice. Int J Mol Sci.

[49] Murphy Dennis J. (2004). Determination of accurate KI values for tight-binding enzyme inhibitors: an in silico study of experimental error and assay design. Analytical Biochemistry.

