# Supplementary material for: Cathepsin S inhibition combines control of systemic and peripheral pathomechanisms of autoimmune tissue injury
Source: Sci Rep. 2017 Jun 5;7:2775. doi: 10.1038/s41598-017-01894-y (PMC5459853; doi:10.1038/s41598-017-01894-y)

Cathepsin S inhibition combines control of systemic and peripheral pathomechanisms of autoimmune tissue injury

Maia Tato, Santhosh V. Kumar, Yajuan Liu, Shrikant R. Mulay, Solange Moll, Bastian Popper, Jonathan N. Eberhard, Dana Thomasova, Arne Christian Rufer, Sabine Gruner, Wolfgang Haap, Guido Hartmann, Hans-Joachim Anders

Supplementary figures

Supplementary figure 1. Biochemical and pharmacological profile of R05459072. (A) Upregulation of invariant chain p10 levels in human B-cells was estimated by p10 ELISA, upon treatment with increasing concentration of RO5459072. The table shows indiviual EC50 of p10 upregulation in B-cells derived from 6 healthy volunteers. (B) Determination of K_M for the fluorogenic substrate Z-Phe-Arg-AMC. (C) Progress curves of hydrolysis of 10 µM Z-Phe-Arg-AMC by 10 nM Cat-S in the presence of no (black dots) or increasing concentration of RO5459072. A linear equation was fitted to the data to determine the slope (solid lines). The bottom panel shows the residuals of the fit to the data. (D) Secondary plot of the slopes from panel B as function of RO5459072 concentration (dots with color coding from panel B). The Morrison equation was fitted to the data to yield K_i = 66 ± 25 pM (solid black curve; with the concentration of catalytically active Cat-S converged to [E]0 = 6.9 nM). The dashed red lines indicate the 95 % confidence intervals of the fit to the data. (E) Pharmacodynamics of R05459072 was determined in C57Bl6 mice with increasing amounts of compound as single dose. Plasma levels of compounds were correlated to the upregulation of p10 in spleenocytes.

Supplementary figure 2. FACS gating strategies used for the different splenic immune cell populations. (A-C) Activated dendritic cells CD11c+CD11b+MHC-II, (D-F) activated macrophages CD11c+F480+MHC-II and (G-H) plasma cells CD138+κ-light chain+

Supplementary figure 3. Plasma IgG isotypes in MRL-(Fas)lpr mice. Plasma levels of IgG1 (A), IgG2a (B), and IgG2b (C) were determined by ELISA at different time points from the initiation (week 11) to the end of treatment (week 19). Data are expressed as means ± SEM (n=8 to 10 in

each treatment group). *p<0.05, **p<0.01 versus vehicle group. ###p<0.001 base line values versus week 19 values.

Supplementary figure 4. Silver staining and CD3 immunostaining of kidney tissue. At the end of the study kidney sections of all mice were stained with Silver (A and C) or for the T cell marker CD3 (B and C). Silver staining was quantified by semiquantitative scoring. The number of intraglomerular CD3 positive cells was counted per glomerulus. Representative images are shown at an original magnification of x400 (scale 25 µm). Data are expressed as means ± SEM. *p<0.05, ***p<0.001 versus vehicle group.

Supplementary figure 5. Kidney cytokine and chemokine mRNA expression levels. Total kidney cytokine and chemokine mRNA expression levels were quantified by real time PCR normalized for the housekeeper 18s rRNA. Data represent mean scores ± SEM. *p<0.05, **p<0.01, ***p<0.001 versus vehicle group.

Supplementary figure 6. Flow cytometry of intrarenal leukocyte subsets. (A-C) From mice of all groups kidney cell suspensions were prepared for flow cytometry using specific antibodies that identify the respective leukocyte subsets as indicated. Data are expressed as means ± SEM (n=8 to 10 in each treatment group). *p<0.05, **p<0.01, ***p<0.001 versus vehicle group.

Supplementary figure 7. Flow cytometric gating strategies. (A-I) FACS gating strategies for identifying different populations of intrarenal immune cells.

Supplementary figure 8. Plasma (auto-) antibody profiles in MRL-(Fas)lpr mice with late onset of treatments. Plasma levels of total IgG (A), anti-dsDNA IgG (B), IgG2a (C), IgG2b (D), IgM (E), and anti-dsDNA IgM (F) were determined by ELISA. Data are expressed as means ± SEM (n=5 to 7 in each treatment group). *p<0.05, **p<0.01 versus vehicle group. ##p<0.01, ###p<0.001 base line values versus week 19 values.

Supplementary figure 9. Markers of lupus nephritis in MRL-(Fas)lpr mice with late onset of treatments. Urinary albumin/creatinin (A/C) ratio (A) and blood urea nitrogen (BUN, B) was determined at different time points as indicated. At the end of the study PAS sections were scored for lupus nephritis disease activity (C) and chronicity (D) (scale 25 µm). Data are expressed as means ± SEM (n=5 to 7 in each treatment group). *p<0.05, **p<0.01 versus vehicle group. Representative images are shown at an original magnification of x400 (E).

Supplementary figure 10. Cathepsin S levels in ISN-RPN lupus nephritis and CKD patients and Cathepsin S /CD68 double immunostaining in healthy human kidney. (A-D) Cathepsin S levels were measured by ELISA in ISN-RPN lupus nephritis (A) and SLE associated CKD patients (B) and correlation analysis for the same (C and D). Dual immunostaining for Cat-S and the macrophage marker CD68 of healthy human kidney biopsy showed Cat-S positivity in CD68 negative tubular epithelial cells. Representative images are shown at the indicated scale 100 µm.

Supplemetary table 1. Slective inhibitory effect of RO5459072 on different cathepsins

S.NO	Cathepsins	IC50 (nM)
1	Human Cat-S	0.1
2	Mouse Cat-S	0.3
3	Human Cat-V	700
4	Human Cat-K	>1000
5	Human Cat-B	>1000
6	Human Cat-L	>1000
7	Human Cat-F	>1000

Supplementary figure 1

A

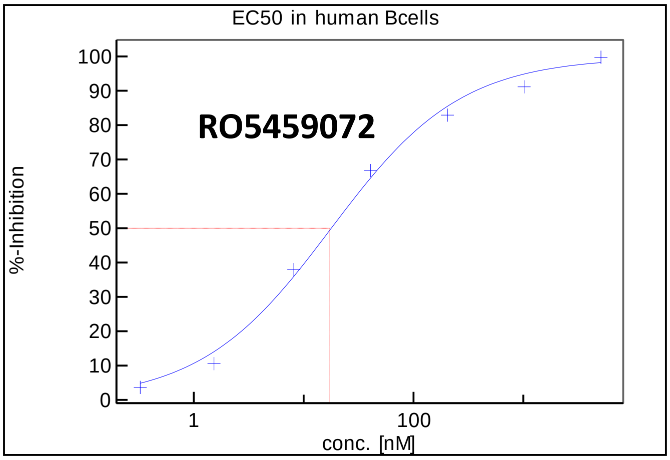

B

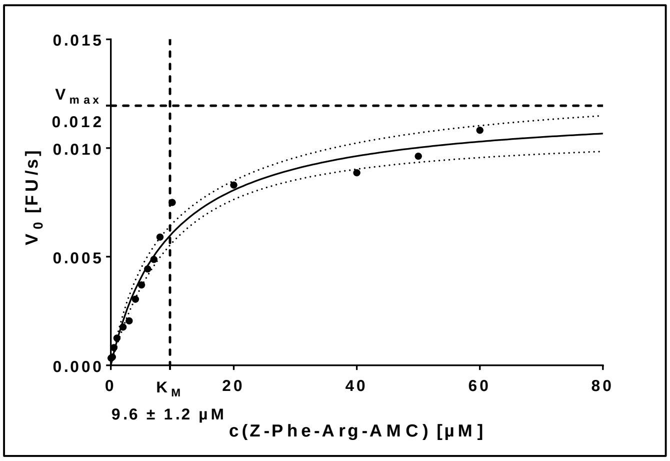

Donor:	D1	D2	D3	D4	D5	D6	Average	std
RO5459072	26.4	15.8	19.1	9.4	12.6	11.5	**15.8 nM**	6.2

C

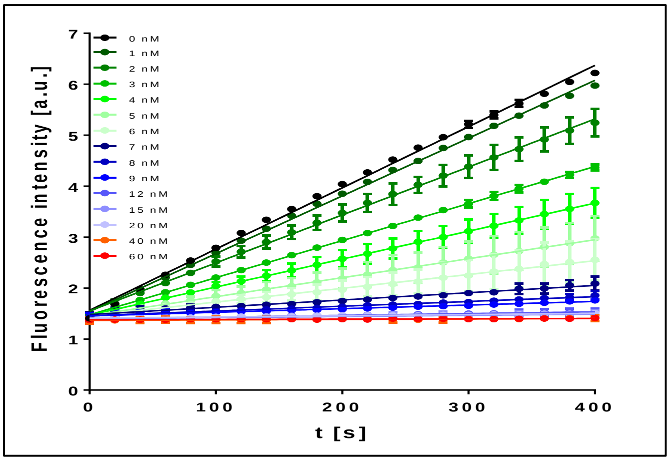

D

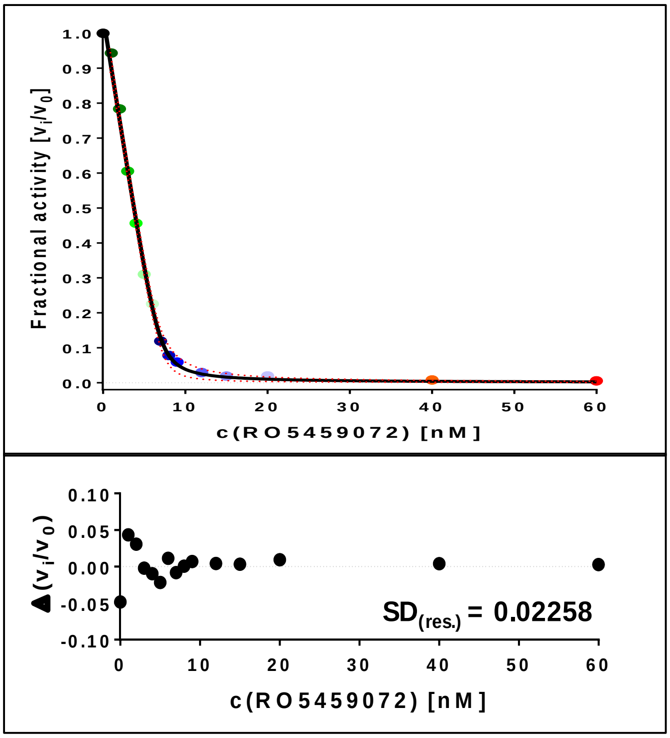

E

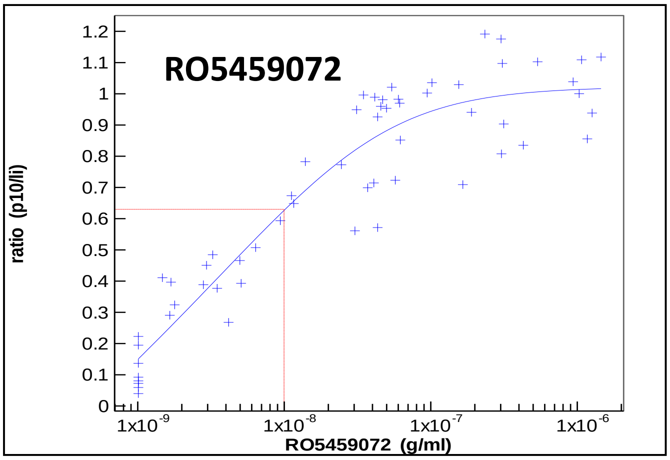

p10/li in vivo:
EC50 = 17 nM

Supplementary figure 2

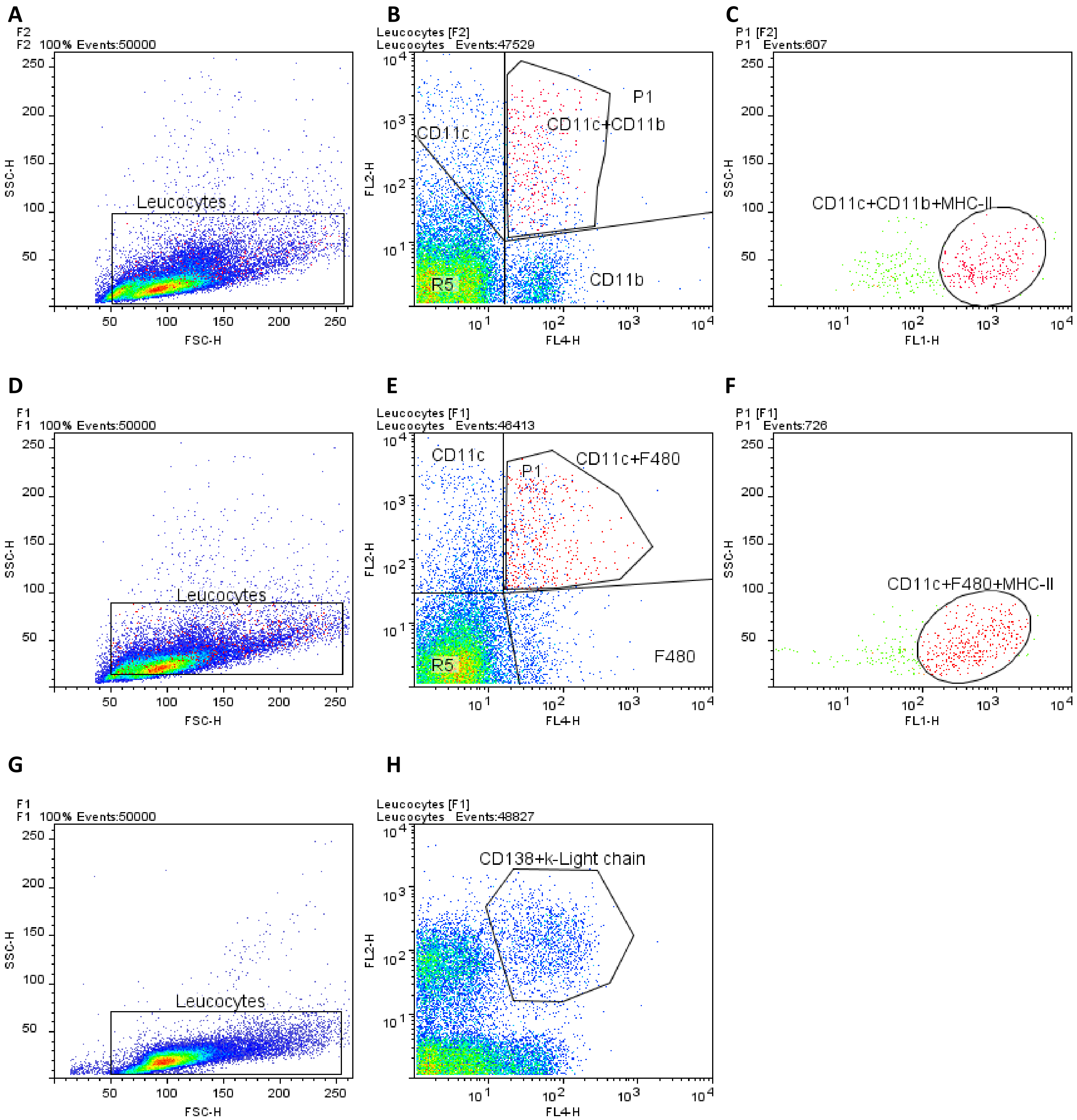

Supplementary figure 3

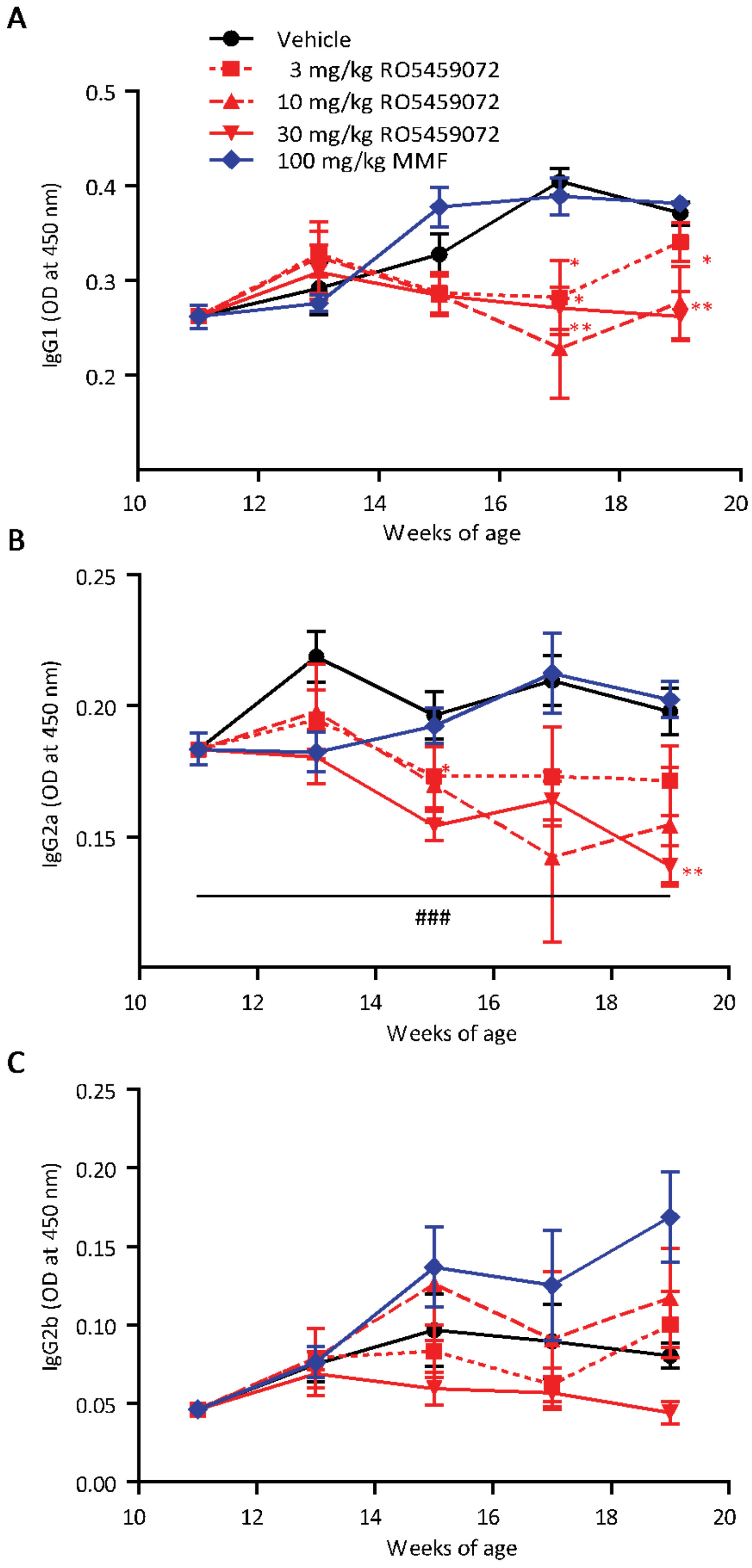

Supplementary figure 4

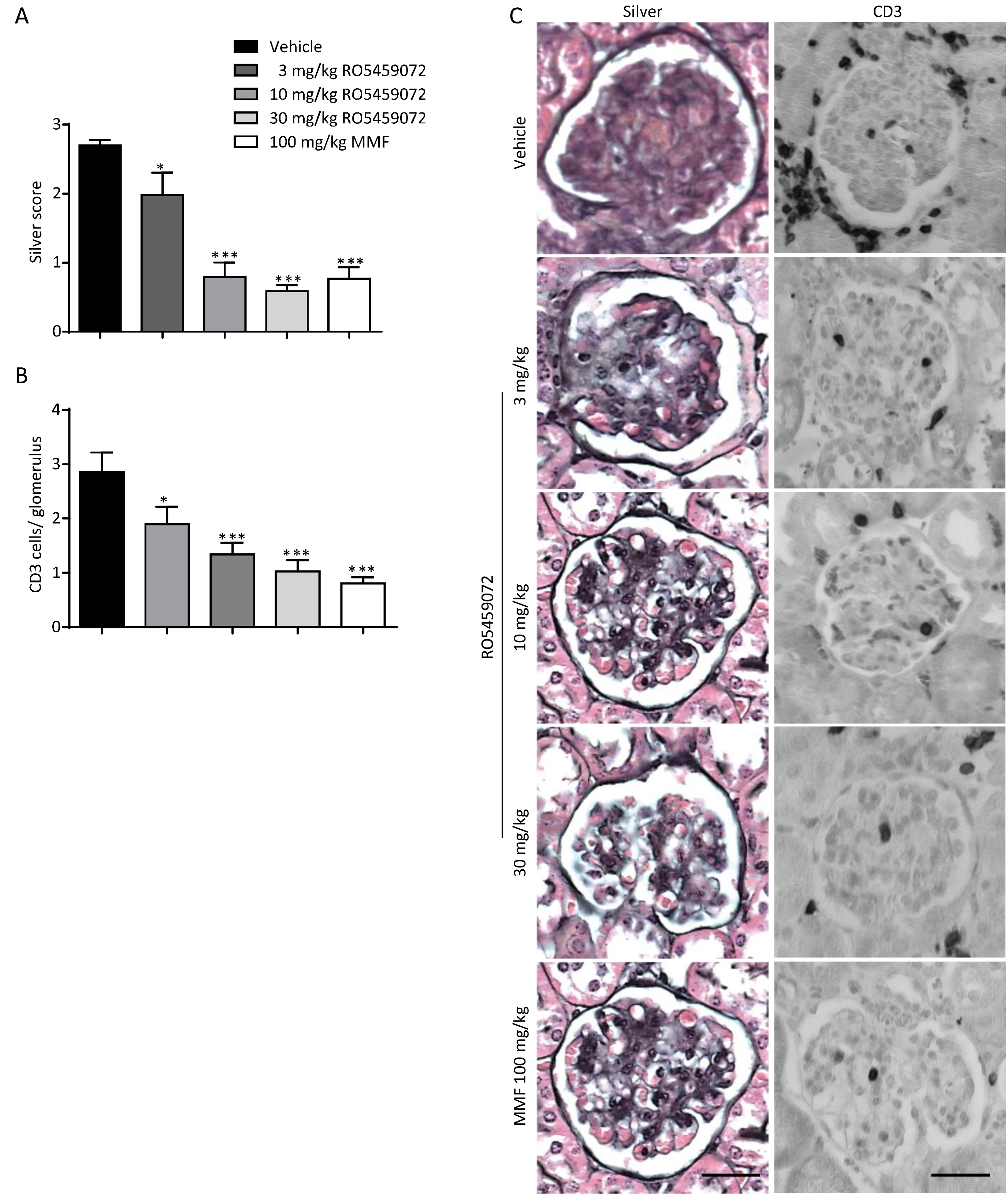

Supplementary figure 5

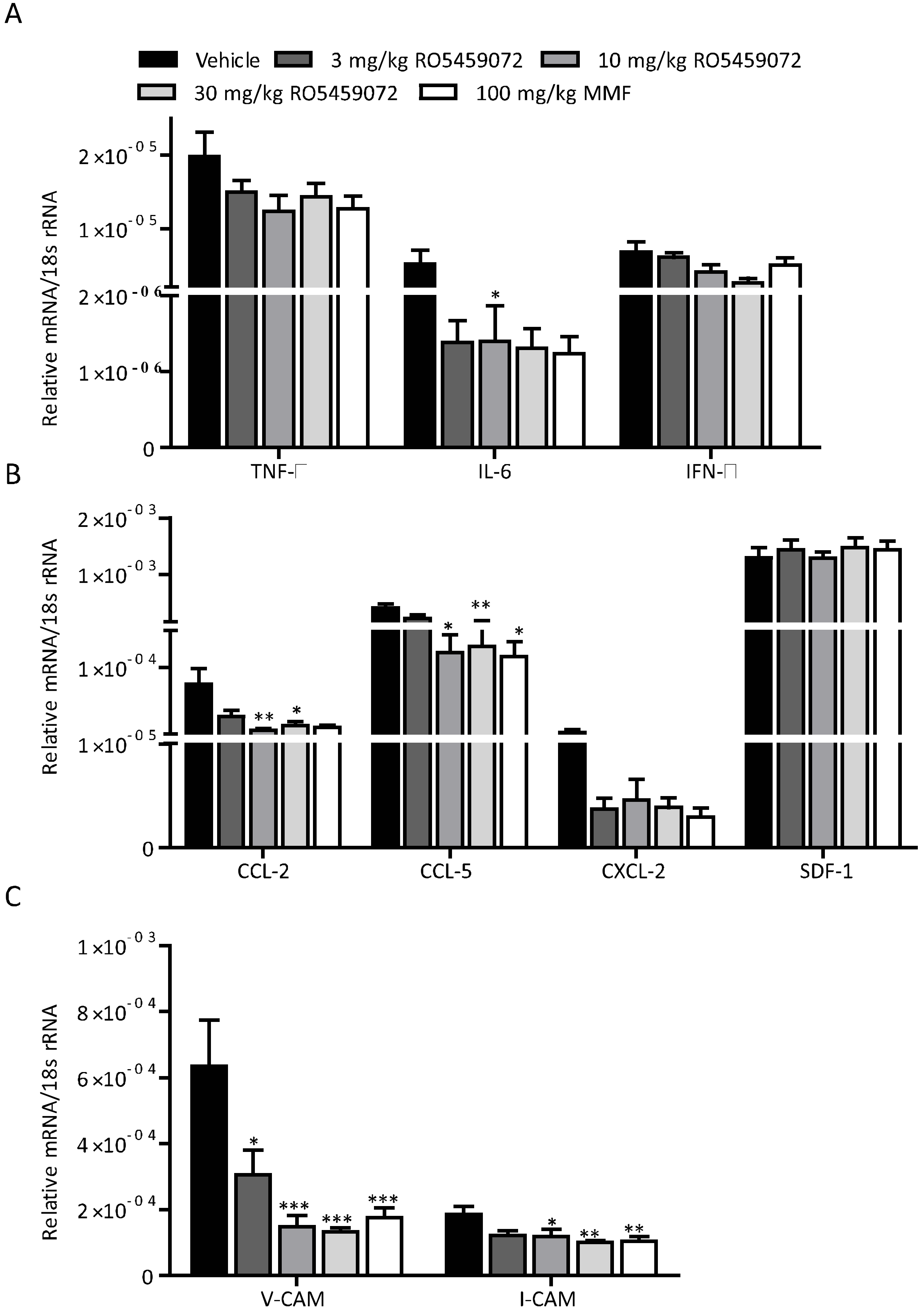

Supplementary figure 6

A

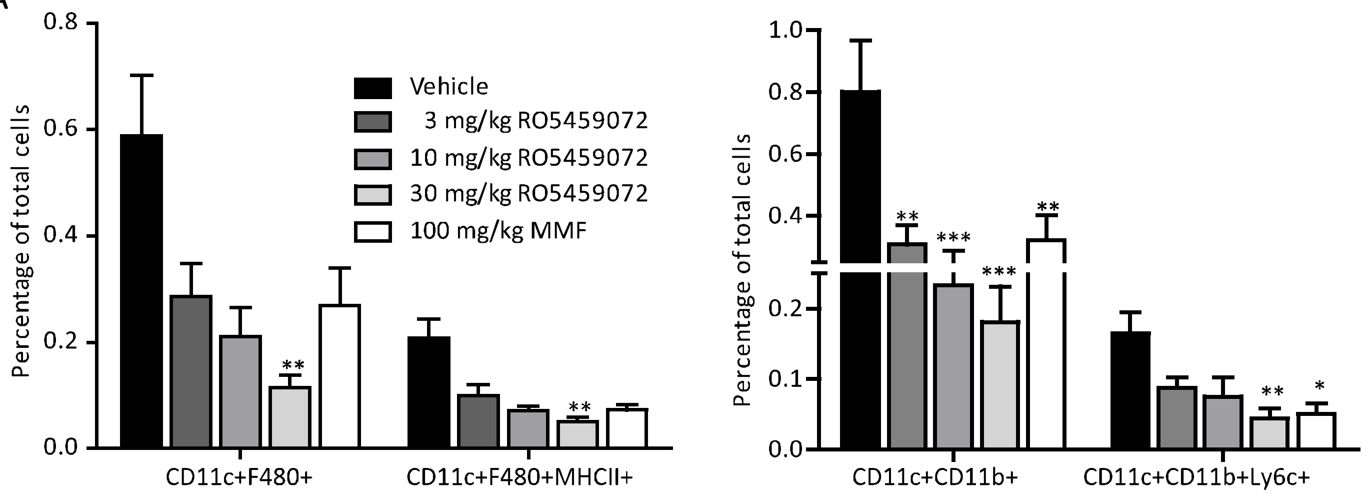

B

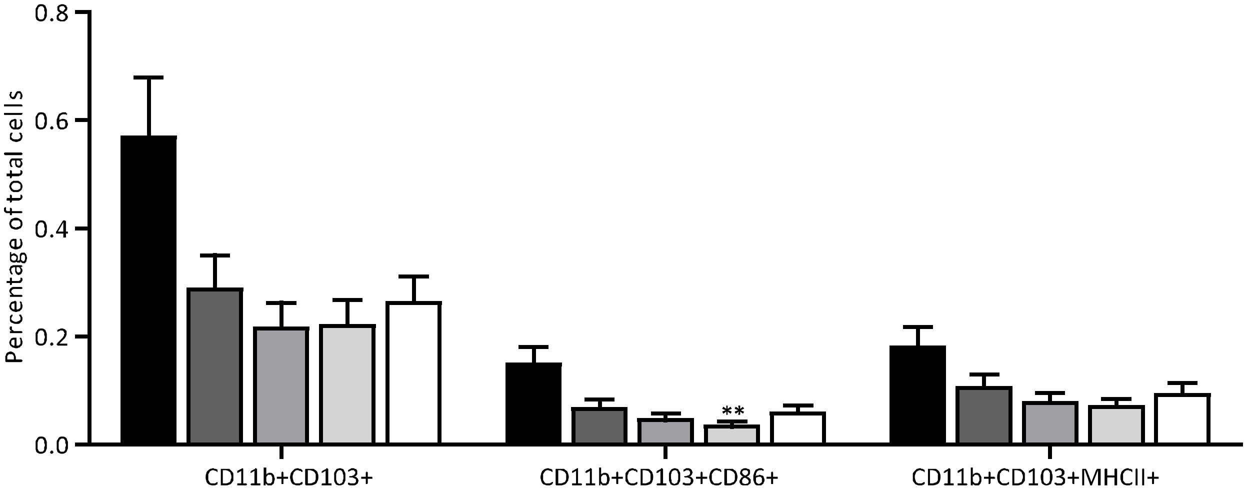

C

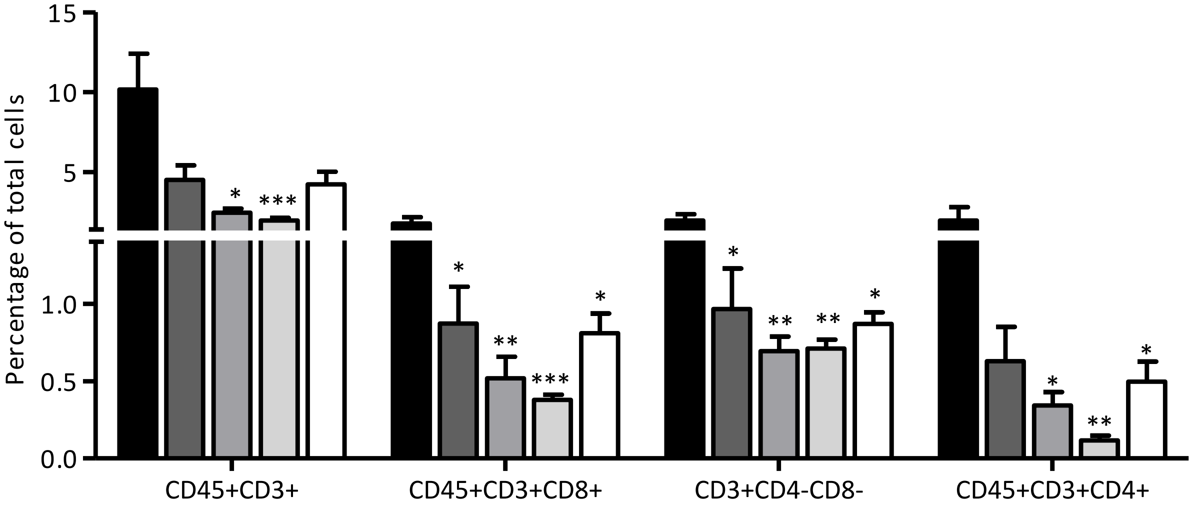

Supplementary figure 7

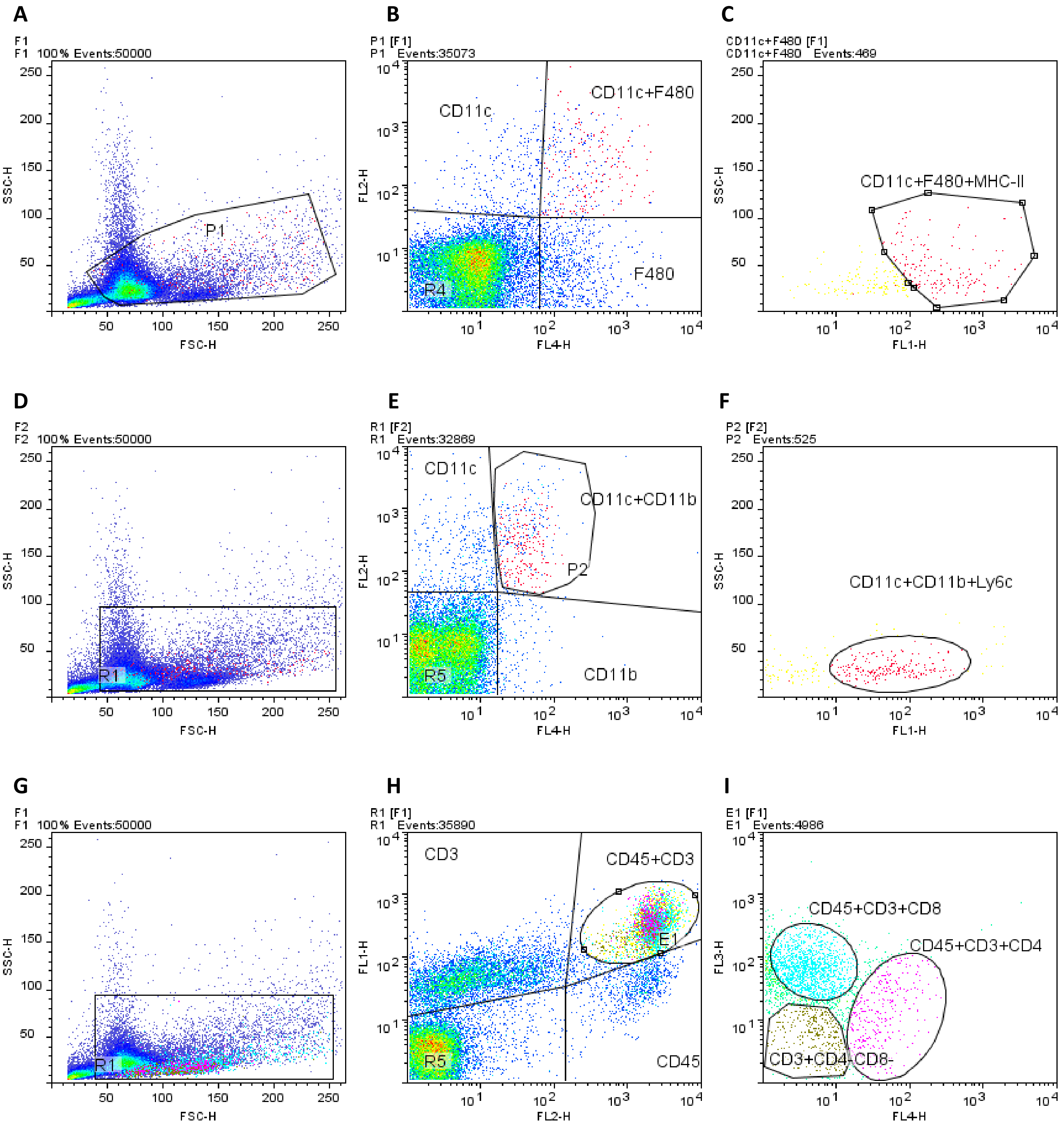

Supplementary figure 8

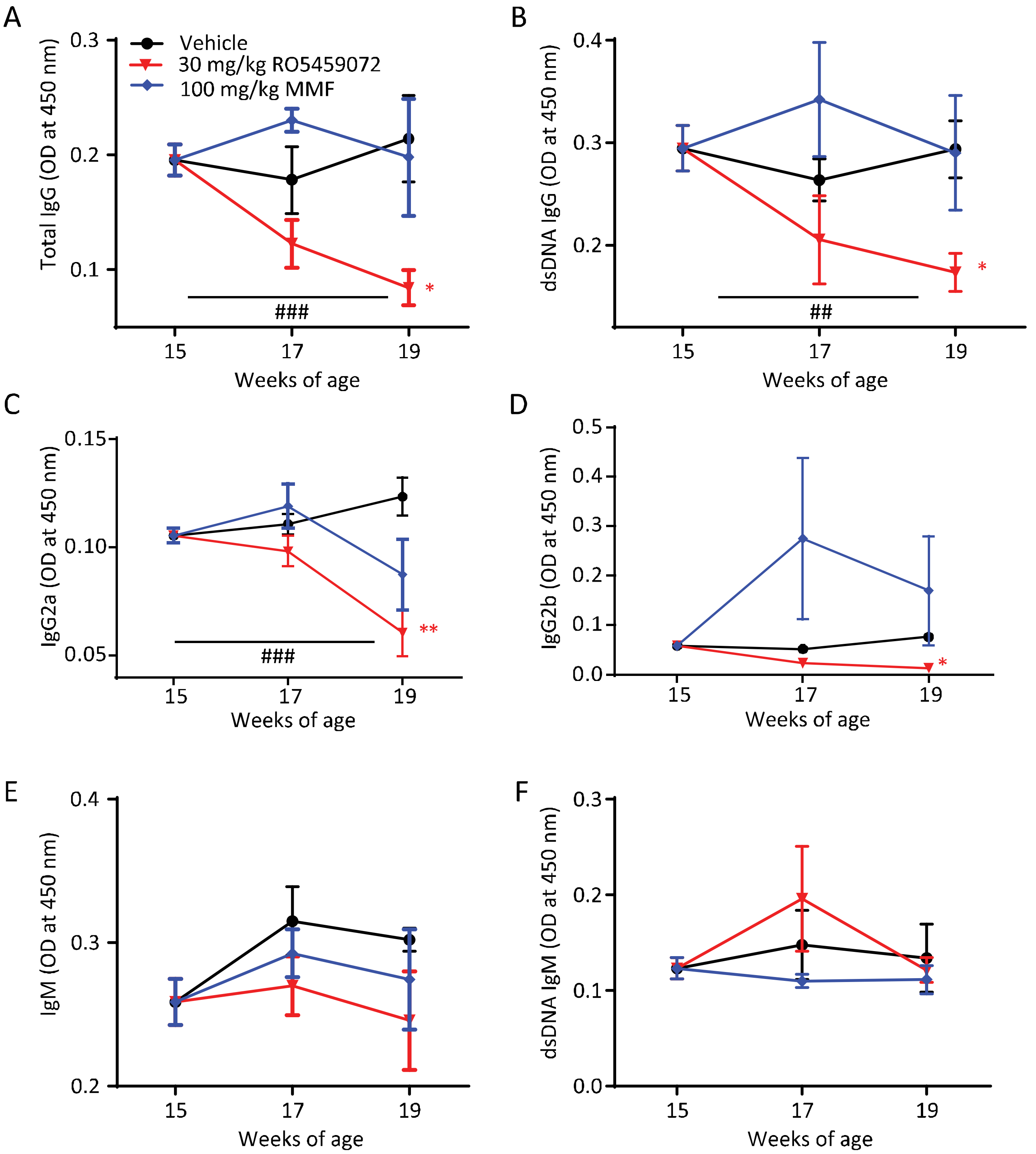

Supplementary figure 9

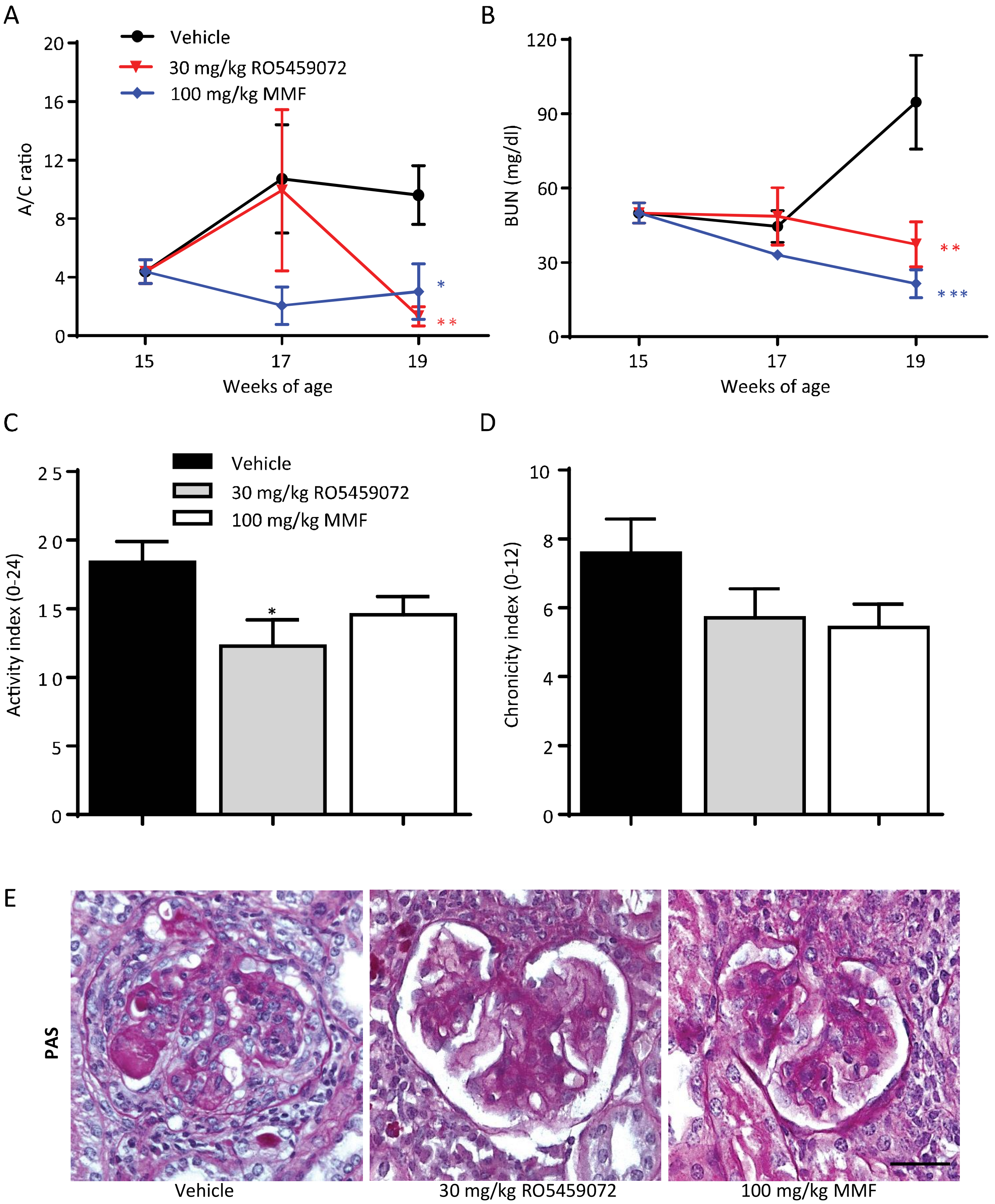

Supplementary figure 10

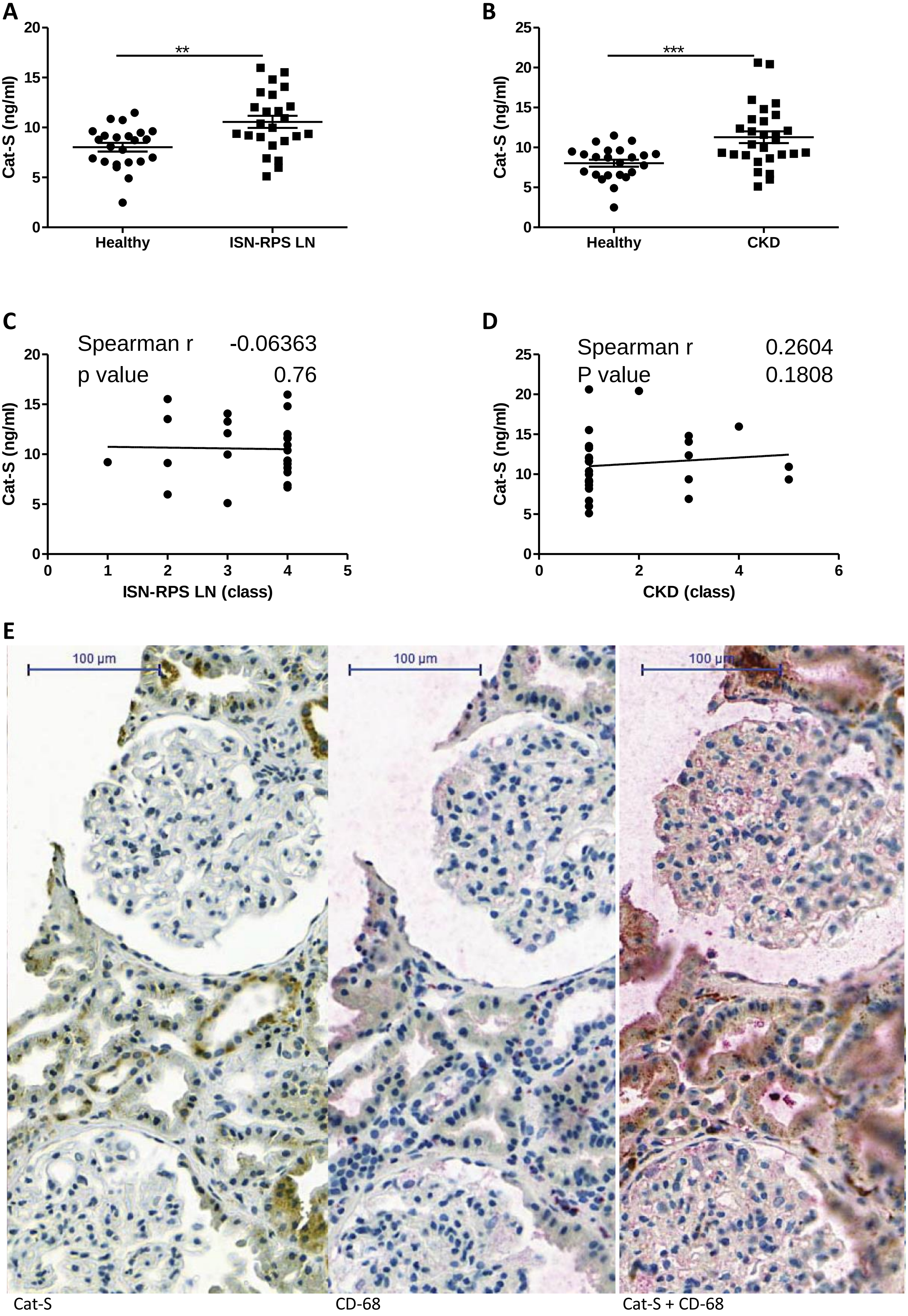

Supplement: Supplementary file 1 — Supplementary information [file 41598_2017_1894_MOESM1_ESM.pdf]
